# Magnetic mediators for ultrasound theranostics

**DOI:** 10.7150/thno.62218

**Published:** 2021-11-02

**Authors:** Arkadiusz Józefczak, Katarzyna Kaczmarek, Rafał Bielas

**Affiliations:** 1 Chair of Acoustics, Faculty of Physics, Adam Mickiewicz University in Poznań, Uniwersytetu Poznańskiego 2, 61-614 Poznań, Poland; 2 Department of Biomedical Engineering, Faculty of Engineering, University of Strathclyde, Wolfson Centre, 106 Rottenrow, Glasgow, United Kingdom

**Keywords:** Ultrasound, Sonosensitizers, Magnetic agents, Theranostics

## Abstract

The theranostics paradigm is based on the concept of combining therapeutic and diagnostic modalities into one platform to improve the effectiveness of treatment. Combinations of multiple modalities provide numerous medical advantages and are enabled by nano- and micron-sized mediators. Here we review recent advancements in the field of ultrasound theranostics and the use of magnetic materials as mediators. Several subdisciplines are described in detail, including controlled drug delivery and release, ultrasound hyperthermia, magneto-ultrasonic heating, sonodynamic therapy, magnetoacoustic imaging, ultrasonic wave generation by magnetic fields, and ultrasound tomography. The continuous progress and improvement in theranostic materials, methods, and physical computing models have created undeniable possibilities for the development of new approaches. We discuss the prospects of ultrasound theranostics and possible expansions of other studies to the theranostic context.

## 1. Introduction

Theranostics has drawn increasing scientific interest over the past decades. The term theranostics was first used in 1998 to describe “the ability to affect therapy or treatment of a disease state” 1. Theranostics (aka theragnosis) is based on the concept of combining therapeutic and diagnostic methods into one platform to improve the effectiveness of treatment. The use of nano- or micron-sized materials enables combinations of modalities and thus provides numerous medical advantages. The growing surge in studies on theranostics is mainly due to progress in materials science, which provides classes of materials designed for theranostic systems. In particular, imaging function has ceased to be only provided by the addition of contrast agents to drug carriers 1.

### 1.1 Ultrasound theranostics

Introducing ultrasound (US) into theranostic treatments can be highly beneficial 2. The clinical utility of US is owed to the short wavelength of ultrasonic waves (on the order of millimeters) and their ability to be focused to a small area. US has already found uses in many biomedical applications. Ultrasonic waves play crucial roles in enhancing drug uptake into skin (sonophoresis) and the destruction of kidney stones (lithotripsy) 3. Moreover, US is effectively utilized in a wide range of cancer treatments, such as high-intensity focused ultrasound (HIFU) and sonodynamic therapy (SDT) 4, 5. US is also commonly used in diagnostic imaging through the techniques known as ultrasonography and US tomography 6-8. Because US is widely accessible, portable, non-ionizing, collects measurements in real time, and has deep tissue penetration capabilities, it is playing an increasingly significant role in theranostics. Additionally, US imaging techniques using various nano- and micron-sized materials can be easily integrated into different therapeutic modalities for guiding and monitoring the therapeutic process and its efficiency what will be described in more detail in subsequent chapters.

### 1.2 Overview of mediators for US theranostics

The effectiveness of US applications can be significantly improved by using mediators, which are called sonosensitizers. Various organic and inorganic micron/nano-sized substances can be used for this purpose 7, 9-11. Microbubble (MB) agents have been extensively used in various US imaging techniques and therapies, such as targeted drug delivery, over the past twenty-five years. MBs have specific advantageous features including high biocompatibility, biodegradability, and easy large-scale fabrication 12. However, their relatively large size, fragility, and short circulation are obstacles in some applications. For instance, to be an efficient US contrast agent, MBs need to be smaller than red blood cells. They also need to have a diameter smaller than 8 μm to allow for transport through small arteries 13, 14. Their microscale size restricts MBs from extravasating out of the vasculature, which limits their ability to deliver cargo in drug delivery applications. Instead, various nanomaterials can be used for more efficient US theranostics. Nanoparticles (NPs) can potentially combine several imaging modalities and therapeutic functions into one multifunctional nano-sized platform for advanced anticancer applications. Over the past decade, it has been demonstrated that NPs exhibit strong interactions with biomolecules both on the surface and inside cells 15. Compared to the organic microsystems conventionally used in US-based biomedical applications, inorganic nanomaterials introduce highly desired characteristics for US theranostics, such as high stability, easy fabrication, and specific acoustic response 7. Various inorganic nanosystems have been proposed in the literature including silica NPs (solid, mesoporous, and hollow morphologies), manganese dioxide NPs, gold NPs, titanium dioxide NPs, carbon nanotubes, magnetic NPs (MNPs), and Prussian blue NPs. Such nanomaterials can be used as contrast agents for contrast-enhanced US imaging (CEUS) and photoacoustic (PA) imaging or as synergistic agents for HIFU, SDT, or US-triggered drug release 7.

**Figure 1:**
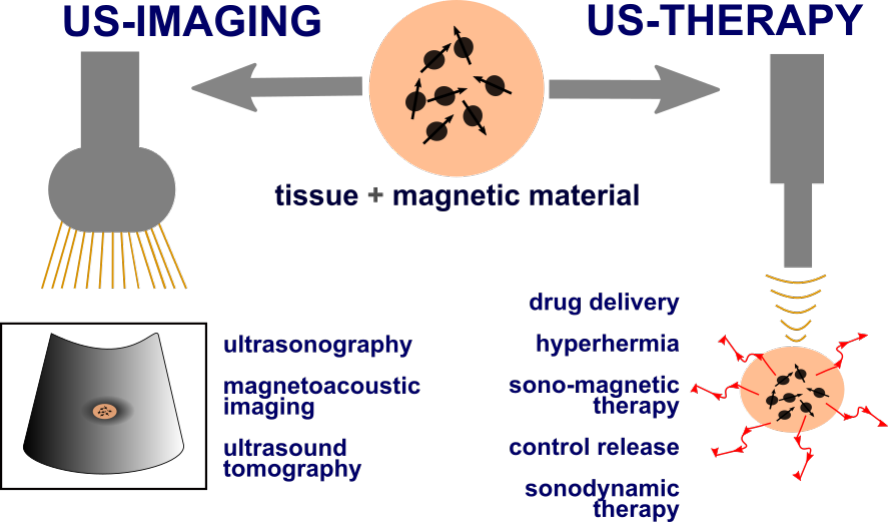
Uses of magnetic materials in US theranostics. Magnetic mediators play crucial roles in fields such as drug delivery and release, US hyperthermia, magneto-ultrasonic heating, SDT, magnetoacoustic imaging, ultrasonic wave generation by magnetic fields, and US tomography as they can act as a heat source, contrast agent, or delivery agent.

For ultrasound theranostics materials with magnetic properties are especially of interest as their incorporation can be highly beneficial for both US-based therapy and imaging. They offer a wide range of properties that make them superior to other sonosensitizing materials. Superparamagnetic iron oxide nanoparticles (SPIONs), i.e., magnetite (Fe_3_O_4_), maghemite (γ-Fe_2_O_3_), and hematite (α-Fe_2_O_3_), possess exceptional superparamagnetic properties, show biocompatibility non-toxicity, biodegradability (they are metabolized by the enzyme heme oxygenase 1 to form blood haemoglobin 16, 17) and can be developed at a very low cost. Their surface can also be easily chemically modified with inorganic molecules, ligands, and polymeric or non-polymeric stabilizers which provide opportunities to use SPION-based agents in various medical applications 17. Magnetic mediators can act as a source of additional heat, or as an imaging contrast agent, can be used for controlled drug release, or can be used in SDT. Additionally, magnetic materials can be incorporated into microbubbles, nanorobots, nanodroplets and therefore change existing non-magnetic mediators into magnetic ones. **Figure 1** illustrates the possible applications of magnetic materials in US theranostics.

A few review articles have already presented the potential of various materials as theranostic carriers 1, 5, 18, 19. Wang *et al.* presented an extended publication survey on the use of iron oxide NPs and derivative materials as theranostic agents 20; however, these agents were not described in the context of US theranostics. The combination of mediators with magnetic properties, magnetism and mechanical ultrasonic waves opens new opportunities for improvements in the design of therapies and multimodal imaging. The most convenient features of each modality can be acquired and merged to diagnose and combat diseases in a more robust way. Therefore, our review emphasizes recent advancements in the field of US theranostics and magnetic materials as mediators. We focus on several subdisciplines including controlled drug delivery and release, US hyperthermia, magneto-ultrasonic heating, SDT, imaging with combined ultrasonic and magnetic fields, ultrasonic wave generation by magnetic fields, and US tomography. We conclude with a discussion of the prospects of the field and possible expansions of other studies to the theranostic context.

## 2. Controlled Drug Delivery and Release

Effective delivery of therapeutics (e.g., small molecule drugs, nucleic acids, genes) to the target site and their precise release from carriers (e.g., MBs, NPs) are crucial steps for achieving successful treatment. To achieve targeted drug delivery, various external stimuli, such as pH, light, ultrasonic waves, acoustic forces, electric fields, and magnetic fields, are commonly used. Alternatively, passive targeting of NPs to tumors by the enhanced permeability and retention effect, active targeting by surface modification of carriers with ligands, or chemical targeting via specific bindings can be used as well 21-24. The application of US for controlled drug delivery and release has attracted increasing scientific attention as it is a relatively cheap and noninvasive modality and US imaging can be easily integrated into theranostic applications.

### 2.1 Magnetic mediators for US-based controlled delivery and release

**Figure 2:**
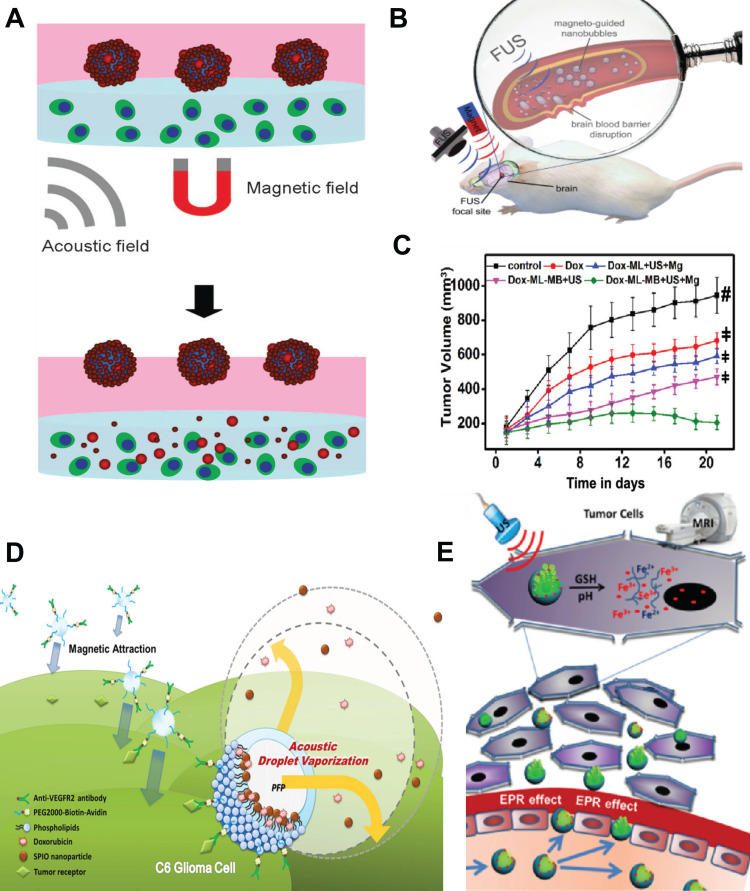
**A.** Schematic illustration of the controlled release of doxorubicin-Poly Lactic-*co*-Glycolic Acid (dox-PLGA) particles from magnetic microbubbles (MMBs) under magnetic and acoustic fields. Adapted with permission from 51, copyright 2016 Springer. **B.** Disrupting the BBB by FUS with magnetically guided theranostic NBs. Adapted with permission from 49, copyright 2015 John Wiley & Sons. **C.** Antitumor efficacy after various treatments with doxorubicin-loaded magneto-liposomes (DOX-ML) and MBs (DOX-ML-MBs) combined with ultrasonic and magnetic (Mg) fields. Adapted with permission from 52, copyright 2020 American Chemical Society. **D.** Scheme of the acoustic droplet vaporization process in anticancer magneto-ultrasonic treatment. Adapted with permission from 35, copyright 2015 Elsevier. **E.** US and MRI bimodal imaging and passively targeted drug delivery using Fe_3_O_4_@PFH@PMAA-DOX microspheres. Adapted with permission from 53, copyright 2017 Elsevier.

Various types of agents containing magnetic materials have been developed over the years; nanodroplets 25, paramagnetic hollow silica nanospheres 26, hollow silica NPs doped with iron 27, 28, lipid-shelled MBs with attached iron oxide NPs functionalized with heparin 29, lipospheres with genes or nucleic acids 30, 31 US-driven nanomotors 32, and magnetically responsive nanorobots 33, 34. Amongst them, also SPIONs are desirable candidates for a theranostic mediator for controlled delivery and release as they possess the ability to function at the cellular and molecular level. Applications of MNPs in US-based delivery and release approaches have been widely discussed. For example, Kariminia *et al.* used magnetite NPs coated with chitosan to investigate US-based rapid antibiotic release in response to a pH change 35. Sengupta *et al.* combined a static magnetic field with low-intensity pulsed US acting on MNPs for enhanced drug delivery 36, 37. This combined treatment increased the efficacy of drug delivery due to alterations in cancer cell membrane potential and permeability and increased cancer cell apoptosis. Authors also showed that the cell membrane permeabilization caused by the integrated action of ultrasonic and magnetic fields facilitated significant internalization of the MNPs into cells. Although use of the MNPs for diagnostics was not mentioned in this study, there exists a potential for their simultaneous utilization as contrast agents for magnetic resonance imaging (MRI). Many successful examples of magnetic carriers as bifunctional agents for MRI-guided focused ultrasound (FUS) and US-mediated drug delivery under imaging guidance, that can improve local drug deposition and distribution, have already been presented in the literature 38, 39. MRI-visible microcapsules with iron oxide-deposited walls have been used for targeted and controlled release of doxorubicin in rat tumor models (**Figure 2A**). The application of FUS and real-time MRI tracking resulted in targeted drug release with a 16‐fold increase in doxorubicin concentration in tumors compared to non-targeted organs 40. Similarly, core-shell MNPs containing doxorubicin have been used as drug delivery systems responsive to US, pH, and magnetic fields 41. In the work of Shakeri-Zadeh *et al*., magnetic nanocapsules loaded with anticancer drugs were magnetically targeted *in vitro* 42. Subsequent ultrasonic sonication of tumors led to controlled drug release and improved therapeutic response. Additionally, the magnetic nanocapsules allowed for imaging of the whole process by MRI. Similarly, liposomes containing MNPs and vascular disrupting agents have been shown to work efficiently *in vivo* as targeted drug delivery agents. Magnetic targeting with thermally triggered release of therapeutics via HIFU has been proven to be effective; however, significant improvement in cancer cure rates has been observed only for the combination therapy 43. Magnetic poly(lactic-*co*-glycolic acid) (PLGA)-based nanobubbles (NBs) were shown to have potential for trimodal imaging (US, MRI, and PA) of breast cancer *in vitro*, while acting as a sustained delivery system for the anticancer drug Herceptin with drug release triggered by low-intensity US 44. Another multimodal approach was described by Niu *et al.* 45. The authors co-encapsulated iron oxide NPs and chemotherapeutic drugs into PLGA-based MBs for US/MR imaging and therapy of lymph node metastases.

### 2.2 Magnetic mediators for US therapy and imaging across the blood-brain barrier

All parts of the human body are not equally accessible to drugs. Delivery of pharmaceuticals to the brain is hampered by the blood-brain barrier (BBB). This is very problematic in the treatment of diseases such as Alzheimer's, Parkinson's, and brain tumors. Because of the BBB, relatively large drug molecules cannot efficiently reach their target sites, which is detrimental to treatment feasibility and effectiveness. The combination of US and magnetic mediators can overcome such difficulty (**Figure 2B**). FUS can transiently permeabilize the BBB and increase passive diffusion of drugs into the brain, while subsequent application of an external magnetic field can actively enhance the localization of drugs attached to MNPs 46. FUS sonication has been used with SPION MBs to simultaneously facilitate BBB opening and enable dual ultrasonic/magnetic targeting of doxorubicin. The distribution of doxorubicin in a rat glioma model was subsequently evaluated by MRI 47. Magnetic NBs in conjunction with US can also be used to open the BBB. NBs can be magnetically guided and monitored by MRI and imaged by US. Because theranostic NBs are capable of delivering therapeutic oxygen and anticancer drugs to brain tumors, they have been proposed for the treatment of central nervous system diseases 48. Similarly, Huang *et al.* manufactured magnetic silica-based NBs by embedding SPIONs into silica-shelled NBs 49. Magnetic guidance was used to increase the local NB concentration for FUS-induced BBB disruption. The accumulated NBs effectively increased BBB disruption efficiency. Another interesting approach to overcoming the BBB was proposed by Carpentier *et al.* 50. The authors presented an implantable pulsed US device called “SonoCloud”, which has already been used in clinical trials. This novel device consists of a 10 mm-diameter US transducer with a resonance frequency of 1.05 MHz that is encased in a biocompatible housing that can be inserted through a burr hole between the skull and brain.

### 2.3 Magneto-ultrasonic approaches to drug accumulation

Lammers *et al.* as an interesting approach to enhancing drug accumulation proposed the use of acoustic forces, e.g., pushing forces generated by HIFU or transcranial US pulses 54. Authors showed that acoustic radiation forces employed for particle guidance concentrated magnetic material in the regions of interest, performing similar to an electromagnet. Additionally they showed good antitumor efficacy after the combined treatment (**Figure 2C**). Release of therapeutics in regions of interest is commonly achieved by mechanical or thermal disruption of their carriers 55-57. However, magnetic liposomes loaded with magnetite and iron-platinum NPs have been used to release drug molecules by strong, short pulses of a magnetic field 58, 59 or alternatively, Liu *et al.* used bursts of ultrasonic waves 60. These not only destroyed the delivery agent, liposomes loaded with MNPs and anethole dithiolethione molecules, but also directly injured tumors.

Other interesting approaches to improving drug accumulation are magnetic droplet vaporization (MDV) and acoustic droplet vaporization (ADV), which were proposed by Wang *et al.* 61. In MDV, an alternating magnetic field was used to activate porous magnetic microspheres (**Figure 2D**). In ADV, droplets were vaporized under HIFU 62. SPION incorporation into acoustic droplets allowed for magnetic targeting and MRI-guided US-triggered ADV. Additionally, ADV of droplets loaded with MNPs and doxorubicin led to cell disruption and drug release. Drug release can also be triggered by low-intensity US. The most common application is called sonoporation, which is a reversible poration of cell membranes. Low ultrasonic wave intensities can effectively release drugs from magnetic NBs by inducing oscillations (**Figure 2A**) 51. Importantly, adjustment of the acoustic intensity allows for balancing of cancer cell porosity and plasma membrane integrity to enhance NB cellular uptake. Increased US intensity increases membrane porosity and permeability, which is beneficial for entry of NPs into cells. The NP uptake efficiencies of cells under US and MB exposure increase with higher US intensity 63, 64**.**

Despite the variability of magnetic mediators and many interesting approaches to enhancing drug delivery and release, the most significant enhancement in cancer treatment efficacy is achieved by the synergetic actions of multiple modalities. Combinations of multiple procedures and integration of drug-loaded magnetic vehicles (e.g., MBs, NBs, microspheres) that have the potential to act as bifunctional US/MR contrast agents leads to the development of novel theranostic platforms that will hopefully allow for US/MR imaging and US-triggered release of anticancer drugs (**Figure 2E**) 53, 65, 66.

## 3. Ultrasonic Thermal Therapy

Hyperthermia is a controlled and deliberately induced moderate temperature rise above 43 °C but not exceeding 45 °C, which is safe only for healthy tissues 4, 67 as cancerous tissues experience permanent and irreversible changes under hyperthermic temperatures. Hyperthermia is commonly used in the clinic for noninvasive heating of muscles and tendons or as an additional support for anticancer therapies 3. Whereas, thermoablation is a high-temperature hyperthermia of 45-50 °C that causes instant cell destruction in a spatially selected area 68. The desired temperature rise in tissues can be achieved by various means, such as ultrasonic waves, alternating magnetic fields, microwaves, infrared waves, and lasers 69-71. However, the primary advantage of ultrasonic waves for tissue heating is that the US beam can be easily focused in a small area to locally deposit high levels of US energy. US focus can be obtained using lenses, concave membrane transducers, phased array transducers, or combinations of all three approaches. The region of ultrasonic focus, known as the focal area, has dimensions usually within several square millimeters 3, 4, 6, 72. The effectiveness of ultrasonic heating depends on many parameters, such as acoustic intensity, transducer frequency, and treatment exposure time. A mismatch of these parameters can result in side effects including discomfort, pain, skin redness, or even burns 3. Regardless, it is possible to achieve desired temperatures without the use of high US intensities or long sonication times. The main core of Chapter 3 is strictly ultrasonic and discusses the ultrasound thermal therapies that can be improved or modified into theranostic by the use of magnetic mediators.

### 3.1 Magnetic sonosensitizers for US hyperthermia

**Figure 3:**
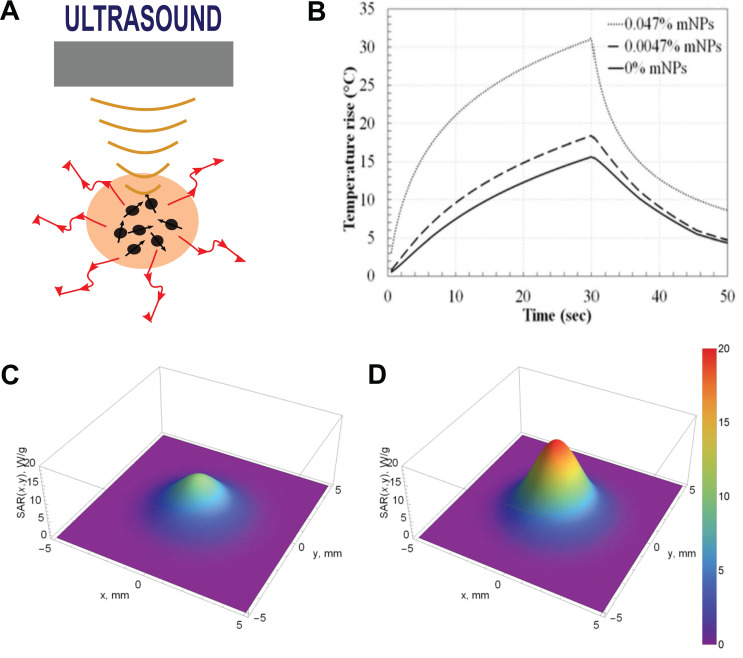
** A.** The concept of sonosensitizer-assisted ultrasonic heating. **B.** Temperature variation over time induced by US in tissue-mimicking phantoms doped with MNPs (14.5 W for a sonication of 30 s). Adapted with permission from 80, copyright 2017 PLOS. Specific absorption rate (SAR) distribution in the focal plane evaluated in a tissue-mimicking phantom **(A)** and a phantom with MNPs **(D).** Adapted with permission from 81, copyright 2018 MDPI.

The effectiveness of ultrasonic heating can be improved by the use of various types of materials as sonosensitizers (**Figure 3A**). Porous silica, gold, and iron oxide NPs have commonly been used 10, 73, 74. The presence of MNPs noticeably improves the thermal effect of US hyperthermia due to additional ultrasonic wave attenuation (**Figure 3B-D**). The interaction between the ultrasonic wave and nanoparticles suspended in the continuous phase of the medium leads to higher attenuation of the ultrasound wave compared to attenuation of the wave in the medium continuous phase alone. The additional ultrasound attenuation consequently leads to the observable temperature increase. Interactions between ultrasonic waves and suspended NPs are explained by differences in the compressibility, density, and thermal properties of the continuous phase (i.e., tissue) and the dispersed phase (i.e., NPs). NPs in an US field tend to pulsate and oscillate. Oscillation leads to the generation of shear waves, and the resulting attenuation is proportional to the difference in density of the continuous and dispersed phases. Pulsation of NPs leads to the generation of thermal waves, and the resulting attenuation is dependent on the difference in thermal properties between the continuous and dispersed phases. Overall, US attenuation, described by the attenuation coefficient, can be expressed as the sum of intrinsic absorption (the combination of ultrasonic attenuation in the NPs and the continuous medium), visco-inertial attenuation, thermal attenuation, and scattering losses 75, 76. US hyperthermia studies performed in tissue-mimicking phantoms indicate that US attenuation increases ~20-150%, resulting in a temperature increase of ~20-70%, when the phantoms are doped with scatterers 77. US attenuation depends on the concentration, size, and US frequency of added scatterers as well as the mechanical properties of the tissue or tissue-mimicking material, such as stiffness 77-79. For example, a comparison study performed on magnetite, silicon dioxide, and Laponite NPs showed that the strongest US attenuation was observed in phantoms doped with magnetite NPs 77.

### 3.2 Ultrasonic heating for theranostic applications

Introducing sonosensitizers to US hyperthermia improves the procedure efficiency; however, what is more important it opens possibilities for further theranostic applications. A theranostic approach combining ultrasonic heating with MNPs was presented by Niu *et al.* 82. The authors used magnetite and perfluorocarbon co‐loaded organic/inorganic hybrid vesicles for US/MR imaging and imaging‐guided HIFU ablation. Similarly, actively targeted theranostic iron oxide NPs have been developed to combine MRI with ultrasonic heating 83. The NPs significantly improved imaging sensitivity for visualizing lung cancer in a rat model and energy deposition efficiency using a clinical MRI-guided FUS system. Chen *et al.* developed a targeted, theranostic iron oxide-based nanocomposite for enhancing the combined antitumor effects of SDT and US hyperthermia during MRI-guided FUS 84. Regardless of the existence of many iron oxide-based theranostic sonosensitizers, new methods for improving thermal therapies using sonosensitizers are constantly being sought, and currently existing approaches are constantly being optimized.

## 4. Magneto-Ultrasonic Heating

The main core of Chapter 4 is strictly magnetic and discusses the combination of magnetic hyperthermia with ultrasound heating and its ability to become a bimodal approach with synergistic features. Such a multimodal technique that combines ultrasonic waves with magnetic fields to generate heat is called magneto-ultrasonic heating. By utilizing the strong points of both modalities (magnetic hyperthermia and ultrasonic thermal heating) and the assistance of magnetic mediators, magneto-ultrasonic heating obtains a better treatment outcome than either of the modalities alone.

### 4.1 Magnetic hyperthermia

MH is a very promising, yet constantly developing, type of thermal treatment that uses MNPs. In comparison to nonmagnetic materials, MNPs are able to transform magnetic energy from an alternating magnetic field into localized heat. There are three mechanisms responsible for heating in MH: eddy current losses, hysteresis losses, and relaxation losses (Brown and Néel modes). When the magnetic moment or the whole magnetic nanoparticle rotates according to the changes of the magnetic field, temperature increases due to friction 69. MNPs can be introduced by direct injection into the tumor area or by intravascular injection. Following systemic injection, MNPs can be accumulated in the desired area by application of an external magnetic field 85. Alternatively, if MNPs are surface-modified with ligands, they can actively accumulate in tumors by binding to specific cancer cells or tissues 86. The advantage of MH over other anticancer procedures is the ability to increase the tissue temperature locally and precisely. Non-healthy tissues with accumulated MNPs are destroyed by the hyperthermia, while surrounding healthy tissues are left intact. There exist other mechanisms able to produce heat; however, in the case of MH, the temperature rise is induced at the nanoscale and the results are visible at the macroscale. This feature is crucial as the major aim of MH is to weaken, sensitize, or even ablate tumors from the inside 16, 69, 87, 88.

MH is a promising approach; however, it has a few issues that need to be considered. Even though the heat caused by the magnetic field is strictly generated in the region containing NPs, hyperthermia systems also produce eddy currents. Most theoretical descriptions of MH neglect eddy currents; however they can result in unwanted, non-selective heating of healthy tissues due to residual heating, so their presence needs to be noticed and discussed. Dutz and Hergt proposed an acceptance limit for the product of frequency and amplitude that should be used in MH to avoid such effects 69. Accumulation of sufficient MNPs in the target tissue is another potential limitation of MH that needs to be addressed. The main concern is that blood flow can wash the MNPs away from the target site. The magnetic force applied to the MNPs needs to be sufficient to overcome the hydrodynamic drag forces exerted on the MNPs by blood flow. Larger NPs exhibit greater tumor accumulation and retention than smaller NPs, which exhibit faster clearance. The limited circulation time of SPIONs decreases their interaction with the applied magnetic field, negatively impacting treatment efficiency 89. Another potential limitation connected to SPION accumulation is the risk of toxicity. Overall, SPIONs are considered to be nontoxic; however, toxicity can arise from accumulation in various sites of the body. A large number of studies have demonstrated that it is possible to accumulate high iron concentrations (≥100 g/mL) in one specific tissue or organ, which may cause toxicity or cytotoxicity. Optimizing the physicochemical properties of SPIONs is a highly effective solution to minimize their immune response and toxicity. Proper surface coatings can stabilize iron oxide NPs and avoid SPION agglomeration. Additionally, surface engineering of magnetite NPs has been shown to reduce oxidative stress and iron homeostasis alteration and, thus, overall toxicity 90. Another issue with MH is that it is not always possible to maintain the desired temperature. In the case of very small treatment areas, the rate of heat production can be slower than the rate of heat dissipation. As these are competing processes, MH is limited to tumors that are not too small in size. One reason for this limitation is the increasing surface-to-volume ratio with decreasing tumor size, which leads to faster heat dissipation into healthy tissue and thus a lower temperature inside the tumor 69. Additionally, heat generation depends on the type of magnetic material used and its spatial distribution in the region of interest. It has been reported that the heating efficiency of maghemite clusters (45-98 nm) is better than that of single NPs (13 nm) because the surface of single NPs cools more rapidly than the surface of clustered NPs 91. Furthermore, while MH works well in liquids (ferrofluids) 98, in more dense media, such as gels or tissues with solid-like structures, its efficiency deteriorates 79. For magnetite NPs, the Brown mechanism dominates for larger particle sizes (>14-17 nm) and in low-viscosity media. The dominant mechanism is also strongly dependent on the hydrodynamic size of the MNPs. The Brown and Néel mechanisms work in parallel; however, the mechanism with the shorter relaxation time dominates, as each MNP chooses the energetically easiest way to change its magnetization 17. Therefore MNPs embedded in tissue are locked in its rigid structure and their ability to rotate is limited 79, 92, 93.

### 4.2 Magnetic mediators for bimodal heating

**Figure 4:**
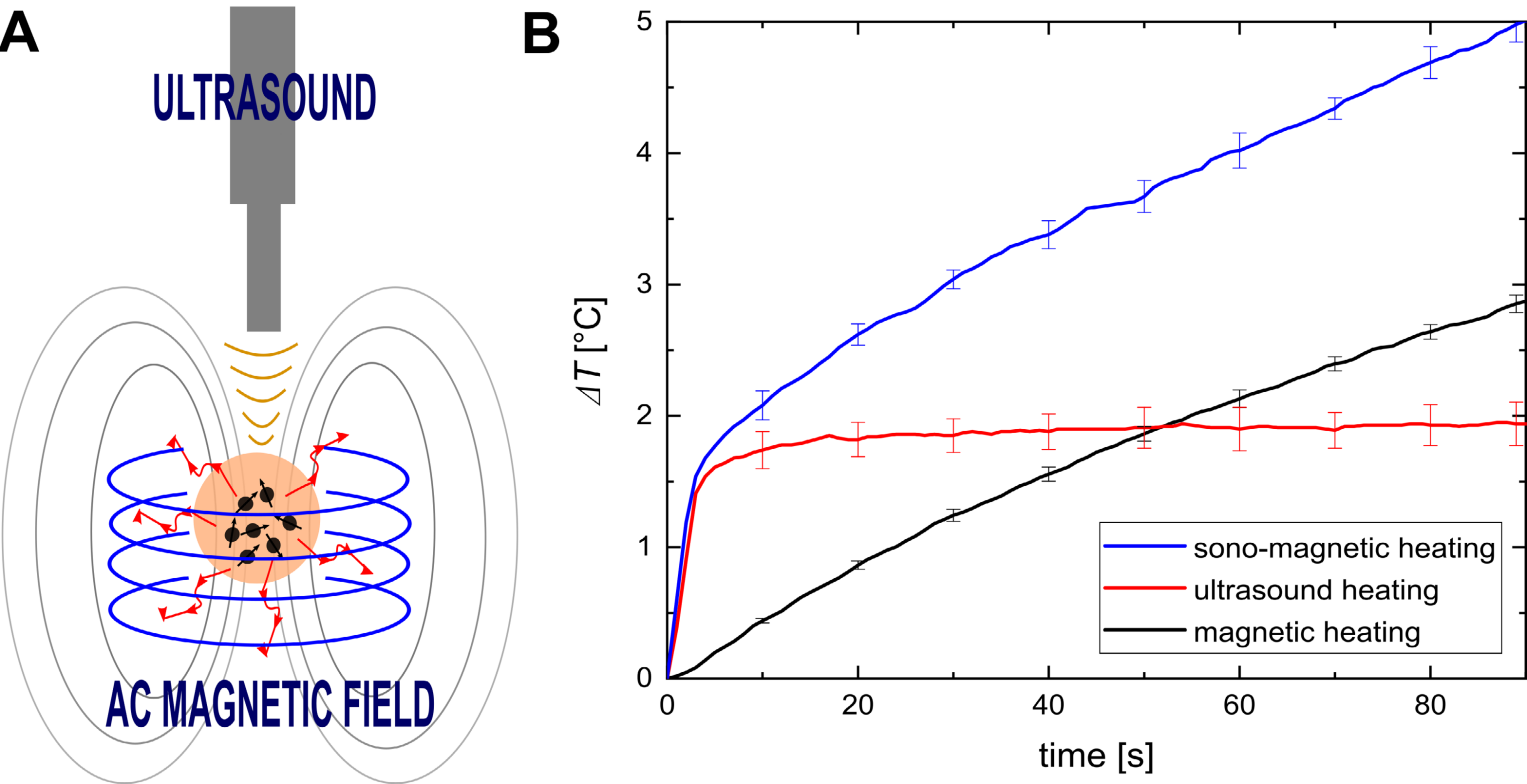
** A.** Concept of magneto-ultrasonic heating. **B.** Temperature increase induced by magnetic, ultrasonic and magneto-ultrasonic heating as a function of time. Adapted with permission from 94, copyright 2020 Elsevier.

The efficiency of MH can be improved by combining it with US (**Figure 4A**). The simultaneous action of the ultrasonic waves and the magnetic field achieve a better heating effect than either heating method alone (**Figure 4B**) 94. Magnetic and ultrasonic heating may work synergistically thanks to bimodal NPs that act as both magnetic mediators and sonosensitizers. Magnetic mediators enhance the efficiency of ultrasonic heating by increasing the overall attenuation of the medium and, subsequently when exposed to magnetic fields, they become the source of heat in MH.

The effects of magnetic and ultrasonic heating seem to be cumulative. It is assumed that the heating improvement observed during magneto-ultrasonic heating results from partial unlocking of the Brown relaxation mechanism by the ultrasonic wave. During bimodal heating, the US sonication increases the temperature and expands the pores of the phantom or tissue, creating more space for Brownian motion. Such bimodal stimulation provides more effective heating, enables more precise control over the heating process, and is more effective than the single heating methods. Therefore, magneto-ultrasonic heating is an innovative and promising approach to treating cancers using low NP concentrations and short exposure times. In the future, we believe this bimodal approach will help to create a new theranostic method suitable for thermal imaging and anticancer therapy. According to the results presented in Figure 9 of 95, the simultaneous use of ultrasonic and magnetic heating resulted in a significant temperature increase. For instance, for the MNP mass concentration of 0.64%, MH contributed only ~6.2% of the temperature increase and US hyperthermia ~73% (with magneto-ultrasonic heating regarded as 100%). Therefore, a synergetic effect was clearly observed. Another advantage of combining two heating mechanisms is that one of the modalities could be chosen to dominate. This would allow for adjusting of the treatment to the depth of the target area. Ultrasonic heating procedures have limited penetration depth, so they could be used to treat subsurface tumors, while MNPs can accumulate in all areas of the body regardless of depth.

Over the years, there has been much interest in the application of bimodal or multimodal thermal treatments 71, 96, 97. A lot of effort has especially been put into combining magnetic hyperthermia (MH) or chemotherapy with photothermal therapy (PTT) 97-99. Various combined treatments allow for a reduction in the amount of potentially harmful (especially in high concentrations) drug dose, substances, sonosensitizers, irradiation time and intensity, and treatment frequency. These small adjustments are highly beneficial as they can reduce the occurrence and severity of negative side effects 3, 97, 100. The work of Espinoza *et al.* showed that the synergistic combination of MH and PTT yielded complete apoptosis-mediated cell death. In solid tumors, the single treatments reduced tumor growth; however, the combination approach resulted in complete tumor regression, heat-induced tumor cell apoptosis and denaturation of collagen fibers at low iron doses, tolerable magnetic field and frequency conditions, and acceptable light doses. This combination thermal therapy is thus promising for tumor treatment with minimal collateral damage to healthy tissues 97. Shen *et al.* developed multifunctional, thermosensitive liposomes encapsulating doxorubicin and loaded with magnetite NPs (DOX-Fe_3_O_4_-TSL) and showed the superior therapeutic effect of chemotherapy-PTT over chemotherapy or PTT alone. DOX-Fe_3_O_4_-TSL significantly inhibited tumor growth while causing no significant damage to normal tissues under NIR laser irradiation 101. The combination of sonoporation and MH was proposed by Merida *et al.* 102. The authors used iron oxide NPs to induce MH and low-intensity US to enhance intracellular delivery during sonoporation. This multimodal treatment demonstrated improved cytotoxicity and reduced cell viability compared with MH alone, which was attributed to the enhanced intracellular drug/NP delivery. The combination of US, MH, and chemotherapy was more effective than either method alone. This work demonstrated that US is a promising noninvasive enhancer for synergistic sono-thermo-chemotherapy. The future challenges of such multimodal approaches will be translating and scaling up experimental setups to meet clinical standards and establishing acceptable values for powers and mediator concentrations for all therapeutic modalities.

## 5. Sonodynamic Therapy

SDT is an anticancer approach that involves the sonication of mediators that become cytotoxic upon US activation. SDT was first introduced by Yumita *et al.* in 1989, where ultrasonic waves were used to activate hematoporphyrin to produce reactive oxygen species (ROS), such as hydrogen peroxide, superoxide anions, hydroxyl radicals, singlet oxygen, and alpha oxygen, to kill tumor cells 103, 104. The probable mechanisms of SDT are based on three main concepts: (i) generation of ROS by a cavitation-activated sonosensitizer, (ii) mechanical damage to cells (e.g., necrosis, apoptosis) caused by the acoustic pressure, and (iii) thermal damage to tissues under ultrasonic sonication 105. SDT is characterized by therapeutic features inherent to US including high tissue penetration depth, high spatial and temporal selectivity, and noninvasiveness.

### 5.1 Magnetic sonosensitizers for SDT

**Figure 5:**
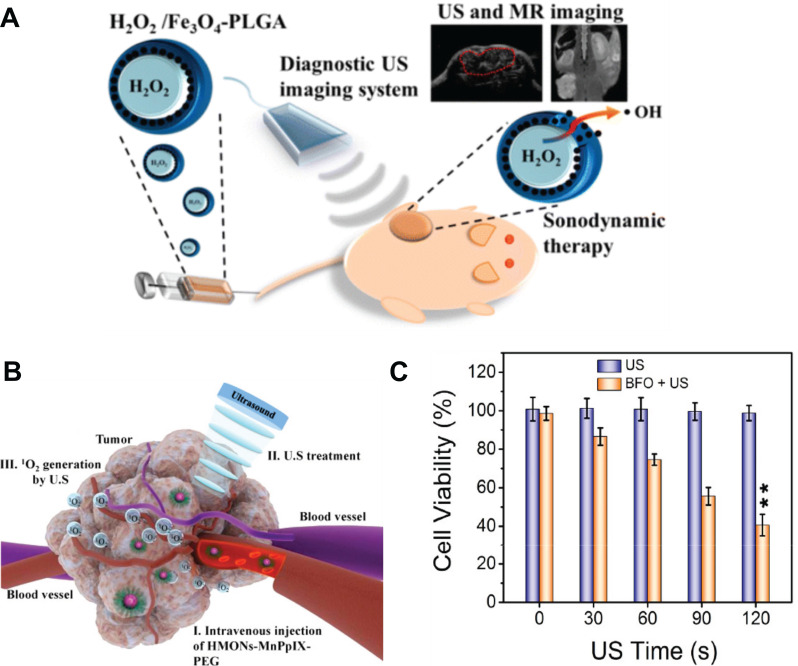
**A.** Schematic illustration of SDT and its mechanism for inducing tumor cell apoptosis using theranostic MNPs composed of magnetite, hydrogen peroxide, and Poly Lactic-*co*-Glycolic Acid (PLGA). Adapted with permission from 126, copyright 2016 American Chemical Society. **B.** Blood transport and tumor accumulation of mesoporous silica NPs loaded with protoporphyrin chelating manganese ions (HMONs-MnPpIX) and its sonodynamic effect for cancer treatment. Adapted with permission from 115, copyright 2017 American Chemical Society. **C.** Relative cell viability under US-enhanced therapy with bismuth ferrite (BFO) MNPs for various US exposure times. Adapted with permission from 116, copyright 2020 American Chemical Society.

Various materials can be used as sonosensitizers for SDT including organic or inorganic micro- or nano-scale compounds 7. Titanium dioxide and natural porphyrin derivatives are the most commonly used materials in biomedical fields as they are nontoxic 106-108. Nanocomposites of titanium dioxide and magnetite have also been used as anticancer drug carriers. The magnetic properties of the carriers allow for their magnetic targeting into tumor cells. The anticancer effects of these NPs have been observed both *in vitro* and *in vivo*, and the synergistic combination of chemotherapy and SDT has been shown to generate outstanding anticancer action 109. In one study, titanium dioxide and gadolinium were combined with a hydrophilic layer and doxorubicin to form a theranostic nanosystem. Under ultrasonic sonication, the combined chemotherapy-STD was shown to be successful *in vitro* and *in vivo*. The advantage of combining SDT with magnetism-based systems is it can allow for MRI-guided multi-mechanism therapy (**Figure 5A**) 110. For example, ultra-small titanium dioxide nanodots doped with iron have been proposed for enhancing SDT of tumors 111. The uses of magnetite NPs without addition of porphyrins or titanium dioxide have also been reported. For example, magnetic liposomes labelled with magnetite NPs and loaded with the SDT sonosensitizer chlorin e6 were delivered using an external magnetic field and activated by US. The great potential of magnetic and US dual-responsive systems for cancer treatment has been demonstrated 112. Bimodal contrast agents composed of iron oxide NPs, ROS-responsive catalase and superoxide dismutase, and hydrogel have also been proposed 113. These hybrid nanogels reacted with ROS within the biological system and generated bubbles to enhance US imaging. Additionally, the gel structure enhanced the reaction of the enzymes with ROS present in pathological tumor tissue. The hybrid nanogels were shown to be efficient US/MR contrast agents for the detection of pathological ROS. Theranostic applications were not directly indicated by the authors; however, the enzymes could potentially be used for therapy. Another multifunctional theranostic agent for imaging‐guided SDT was fabricated by encapsulating sinoporphyrin sodium chelating manganese ions into nanoliposomes 114. Cell and animal studies demonstrated that this agent produced singlet oxygen upon US exposure, which killed cancer cells and significantly reduced tumor growth. The potential of mesoporous silica NPs as carriers of magnetic materials and sonosensitizers for MRI-guided SDT of cancer has also been recognized 115. Mesoporous silica NPs were loaded with porphyrin chelating paramagnetic manganese ions. The high porosity of the NPs was shown to be advantageable for sonosensitizer delivery, as it enabled high loading of the organic sonosensitizers. **Figure 5B** schematically presents the tumor accumulation of the NPs and their sonodynamic effect on tumors. The NPs demonstrated controllable biodegradation, high biocompatibility, and high SDT efficiency for inducing cancer cell death *in vitro* and suppressing tumor growth *in vivo*. Theranostic carriers based on mesoporous silica NPs have also been proposed by Chen *et al.* 84. ROS generation and ultrasonic heating were enhanced by the presence of graphene nanosheets and iron oxide NPs in the silica nanocarriers. For precise therapeutic efficiency, a magnetic field was used to accumulate the MNPs in the tumor site. The authors indicated that the combination of SDT, US hyperthermia, and magnetic targeting led to damage of both superficial and deep regions of tumors. Less common materials have also been used for efficient SDT. For example, bismuth ferrite oxide NPs were designed for chemotherapy-SDT guided by MRI, computed tomography (CT), and fluorescence imaging (**Figure 5C**) 116. Gorgizadeh *et al.* showed that exposure of cancer cells doped with nickel ferrite carbon composites to low-intensity US resulted in heat and ROS production followed by cell death 117. Liang *et al.* designed nanocomposites of holo-transferrin, protoporphyrin, and manganese dioxide nanocrystals that can cross the BBB for MRI and SDT 118. Obvious suppression of tumor growth was demonstrated in a tumor model, and the designed nanocomposites exhibited high biocompatibility and biosafety *in vivo*.

### 5.2 SDT combined with other modalities

To achieve more efficient results, SDT can be combined with other therapeutic modalities, such as US-triggered drug delivery or phototherapy. For example, Gorgizadeh *et al.* showed that the combination of US (SDT) and laser (photodynamic therapy) exposure in the presence of injected manganese ferrite composites led to improved cytotoxicity and deep tissue necrosis in a mouse melanoma tumor model compared with either treatment alone 119. Sheng *et al.* proposed the combination of US and a magnetic field for targeted SDT and drug delivery using magnetic MBs loaded with an anticancer drug and sonosensitizer. This concept led to a 48.3% reduction in tumor volume in mice relative to an untreated control group 120. Beguin *et al.* proposed a magneto-ultrasonic device for chemotherapy-SDT 121. The designed probe enabled co-alignment of magnetic and ultrasonic fields for targeting, which resulted in increased drug deposition in a tissue-mimicking gel phantom of a 40% and an 37% reduction in tumor volume *in vivo* relative to the pre-treatment volume. An interesting combination of MH and SDT can be found in the work of Zhang *et al.* 122. The authors manufactured hollow magnetite NPs with hematoporphyrin embedded in their cavities and a surface coating of polydopamine and polyethylene glycol. Under an alternating magnetic field, these MNPs acted as a source of heat when dispersed in aqueous solution, gel, or bovine liver. Tumor growth in mouse models was inhibited due to the synergetic combination of MH and SDT that was performed with low-intensity US with proven efficiency for ROS generation. Elevated oxygen saturation within tumors was observed by PA imaging, which indicated that the NPs could catalyze the generation of oxygen from endogenous hydrogen peroxide. Tissue oxygenation is an important parameter for predicting tumor response to therapy, and therefore determines treatment outcome. Another approach combines SDT and local drug delivery of titanium dioxide-encapsulated magnetite NPs loaded with doxorubicin 123. The combined pH-induced release of the anticancer drugs and subsequent ROS generation by SDT resulted in superior therapeutic efficiency *in vitro* and *in vivo*. This result was achieved due to the enhanced cell membrane permeability and increased uptake of doxorubicin. Recently, Wang *et al.* described a novel modality combining chemodynamic therapy (CDT) with PTT 124. The results from *in vitro* studies showed remarkable CDT/PTT efficacy, with complete apoptosis of the cancer cells. Gong* et al.,* in turn, proposed a novel noninvasive modality combining low-intensity US, sonosensitizers, SDT, and photodynamic therapy 125. The synergistic CDT-SDT relieved hypoxia to promote ROS production and enhance tumor toxicity.

## 6. Magnetoacoustic Imaging

Ultrasonography is one of the most commonly used imaging methods in the clinic. It is characterized by high sensitivity, broad accessibility, portability, low cost, sufficient spatial resolution, and poor imaging contrast. Longtime use of US as a diagnostic tool has directly shown that spatial resolution and penetration depth are competitive and dictated by the choice of US frequency. Higher frequencies provide better spatial resolution but lower penetration depth. 3 MHz US penetrates tissues up to 1.6 cm, and 1 MHz US is appropriate for imaging tissues at depths of 2.3-5.0 cm 127. Similar dependencies are present in therapeutic US. Therefore, when different therapeutic and/or diagnostic modalities are combined, the limitations of US seem to be the most prominent and difficult to overcome. Hard tissues, such as bones, have much higher impedances (~8 MRay) and attenuation coefficients (~10 dB/cm/MHz) compared to soft tissues (~1.6 MRay, average 0.5 dB/cm/MHz), which has a detrimental impact on the overall performance of US, including imaging resolution, penetration depth, and therapeutic efficacy 128. To improve the overall accuracy and sensitivity of US-based imaging, various US contrast agents have been introduced, which have subsequently contributed to the development of new imaging approaches.

### 6.1 Contrast agents for US-based imaging techniques

MBs are micron-sized microspheres filled with gas that are used in CEUS. They improve the quality of US imaging due to differences in the compressibility, density, and acoustic impedance of the surrounding medium and the MBs, which also have nonlinear behavior in the acoustic field. CEUS has an improved ability to characterize and detect tissue lesions under 1 cm in size, provide information on vascularization patterns, and exploit differences in blood flow characteristics between healthy and pathological tissues 129. From a theranostics point of view, it would be more advantageous to use NPs than MBs for CEUS 130. However, the typical wavelengths of clinically used US systems are several orders of magnitude larger than NPs, and single NPs are not expected to yield strong enough signals for reliable detection by B-scan. NPs are weak contrast agents for ultrasonography as they are too small to backscatter ultrasonic waves at a detectable level 131. Regardless, it is still possible to successfully use MNPs for US imaging and US-based theranostics. MNPs on their own and incorporated into MBs can be used as theranostic contrast agents to, for example, increase the therapeutic efficacy of US hyperthermia. Several imaging technologies are based on the use of functionalized SPIONs as targeted agents for molecular imaging and site-localized drug delivery 132. One approach is MRI-guided HIFU. However, for this approach, the US equipment needs to be adapted to function in strong (several T) magnetic fields, and the treatment itself needs to be delivered in an electromagnetically shielded room. These limitations, along with the high maintenance requirements of MRI systems, make MRI-guided HIFU systems bulky and relatively expensive. Hence, there exists a clear need for the development of alternative, noninvasive methods for imaging and temperature monitoring 133.

### 6.2 Magnetomotive US imaging

**Figure 6:**
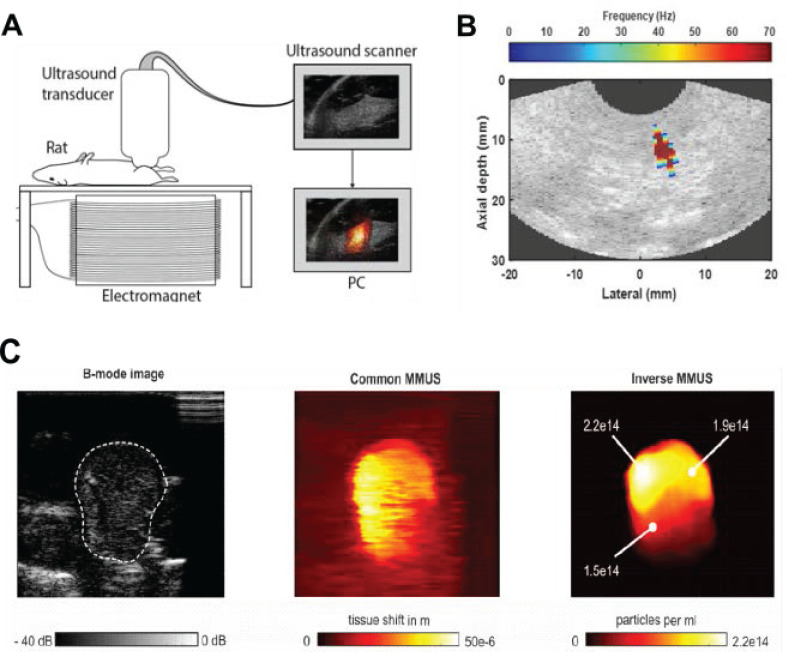
**A.** Magnetomotive Ultrasound imaging system (MMUS). Adapted with permission from 131, copyright 2017 Springer. **B.** Localization of MNPs inside the body by MMUS. Adapted with permission from 145, copyright 2016 Springer. **C.** B-mode image of a tissue-mimicking phantom with MNPs (left), common MMUS evaluation of raw US data (center), and evaluation according to the inverse MMUS algorithm (right). Adapted with permission from 140, copyright 2019 De Gruyter.

A relatively new technique, called magnetomotive ultrasound imaging (MMUS), that combines the magnetic properties of MNPs with US imaging has been demonstrated by Oh *et al.* 134. MMUS induces movement of MNPs by applying a time-varying magnetic field. Excitation of SPIONs with a low-frequency (4-16 Hz) alternating magnetic field causes them to oscillate, which subsequently creates a low-frequency NP-laden tissue vibration that can be imaged using US. This movement allows for identification of tissues that have taken up SPIONs from their surroundings (**Figure 6A**) 135-137. Several variations of MMUS have already been developed, including pulsed MMUS 138, backward MMUS 139, inverse MMUS 140, magnetomotive optical Doppler tomography 141, and MMUS to create tomographic images (**Figure 6B-C**) 142. Frequency and phase tracking MMUS algorithms have been successfully evaluated in simulations, *in vitro*, *in vivo*, and postmortem in rat models 131, 135, 143, 144.

The advantages of MMUS compared to CEUS are its ability to recover stiffness information, accuracy in localizing and identifying small inclusions such as lymph nodes, and the opportunity to reuse MNPs after imaging for further theranostic approaches (e.g., MH, targeted molecular imaging). In comparison, the reuse of MBs after imaging is strongly limited by their short circulation time of several minutes 14. Theranostic applications using MMUS have been proposed by Hadadian *et al.* 146. The authors combined MMUS with US thermometry and MH. US thermometry was used to monitor the temperature changes during MH, and a single MNP acted as a contrast agent for both MMUS and MH. The proposed system successfully localized MNPs, provided real-time 2D temperature maps during MH, and qualitatively predicted the temperature distribution in MNP-laden regions. Because SPIONs are used in MMUS and tissues have temperature-dependent elastic properties, MMUS can also be used for thermometry during MH 147. Various electromagnetic-acoustic techniques, such as magnetomotive photoacoustic imaging (MMPA), have also been proposed for theranostic applications. MMPA uses MMUS to suppress undesired PA signals from background tissue and therefore improve imaging contrast. Liposomal NPs that possess optical absorption in the NIR region and superparamagnetic properties have been developed as MMPA contrast agents 139. MMUS and MMPA can also be used to estimate MNP distribution, MNP displacement in response to a magnetic field, and as dual-contrast sensing techniques for elasticity and viscosity characterization during medical procedures 148. This hybrid magneto-ultrasonic approach provides excellent theranostic potential for the guidance of targeted therapies and treatments 137.

### 6.3 Contrast-enhanced magnetomotive imaging

Despite the many advantages of MMUS, it also has some limitations. The main difficulty with the MMUS approach is that the ability of MNPs to move depends on the viscoelasticity of the surrounding media. The amplitude of MNP movement and tissue displacement are decreased in stiffer tissues, such as tumors, in comparison to surrounding healthy tissue (as in MH). However, researchers have already proposed ideas for enhancing stiffness sensitivity using contrast-enhanced MMUS approaches. In this novel solution, MBs loaded with SPIONs (SPION-MBs) are used instead of MNPs as the US contrast agent 149. Modelling has shown that SPION-MBs are capable of inducing larger tissue displacements (up to 2.3 times larger) than SPIONs alone. As tissue stiffness reduces the amplitude of NP movement, the increase in tissue displacement achieved with SPION-MBs could be beneficial for imaging sensitivity. The authors believe that their approach is able to offer additional diagnostic and perfusion information that could be especially beneficial for colorectal cancer staging 149. Despite the known fragility of MBs, coating them with SPIONs is highly beneficial as the shell rigidity increases several times compared to uncoated MBs 14*.*

There also exists a potential for the proposed contrast-enhanced MMUS technique to be utilized in theranostics. MBs can be combined with anticancer drugs, other therapeutics, and genes, as we presented in Section 2. Subsequently, high-intensity US pulses or MH could be used to destroy the MBs and precisely release the therapeutics.

## 7. Ultrasound Tomography

In the actual age of computerization, scientists are striving to perfect methods for reflection- and transmission-based US imaging that have been used in clinical practice for decades. Specifically, they are focusing on two basic concepts: (i) ultrasonic projection, which is analogous to roentgenography, and (ii) US transmission tomography, which is analogous to CT 150. During ultrasonic measurements, various acoustical parameters that characterize the structure of a sample can be obtained, including the mean amplitude of the ultrasonic wave transmitted through the sample, the runtime on the transmitter-receiver path, and the change in ultrasonic wave frequency after transmission through the sample. A tomographic scanning setup is able to gather these parameters from different directions around the sample. Subsequently, distributions of those local acoustic parameters can be reconstructed from the collected data, which renders the possibility of recreating the internal structure of a sample to generate cross-sections and temperature images 151.

### 7.1 Magnetic mediators for US tomography

**Figure 7:**
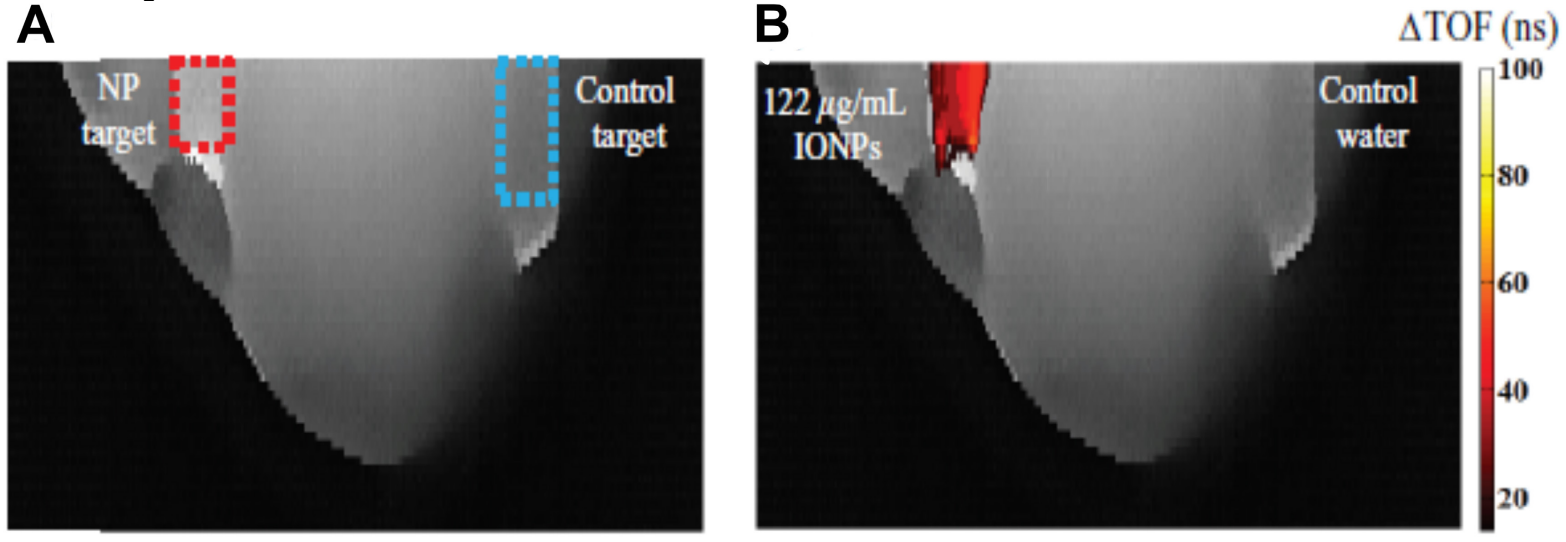
Images of time-of-flight projections in an agar-based breast tissue phantom without **(A)** and with **(B)** injection of MNPs. The dashed squares indicate the target regions into which equivalent volumes of SPIONs or control water were injected. Adapted with permission from 152, copyright 2017 IOP Publishing.

SPIONs can be used as effective “sound speed-based” contrast agents, which are potentially useful for US tomography. When NPs reside in a media, US velocity and attenuation may be altered depending on the NP properties. Such alterations are already used for enhancement of US hyperthermia (see Section 4) or can be used for US imaging, as Perlman *et al.* have shown in their work 152. This imaging modality is based on the consistent relationship between SPION concentration and the speed of sound increase. The authors showed a substantial improvement in the contrast-to-noise ratio, which enabled detection of the NP location. **Figure 7** presents US-CT images with the noticeable effect stemming from the presence of SPIONs.

**Figure 8:**
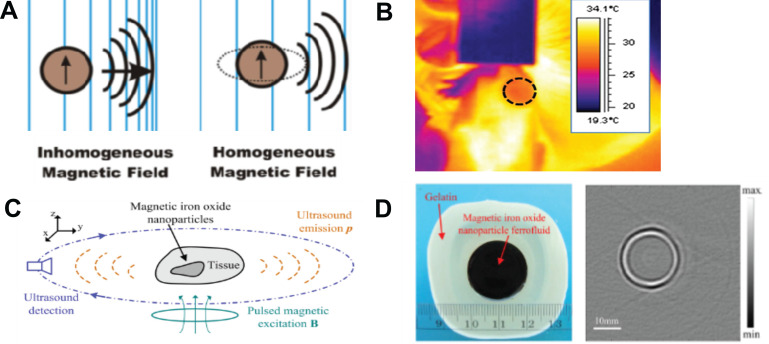
**A.** Generation of ultrasonic waves using MNPs. Adapted with permission from 153, copyright 2016 American Chemical Society. **B.** An image from thermoacoustic tomography. Adapted with permission from 155, copyright 2016 SPIE. **C.** Schematic diagram of MNP imaging and **D.** photograph of a phantom containing an MNP target and its reconstructed tomographic image. Adapted with permission from 58, copyright 2012 AIP Publishing.

Aside from being speed-based contrast agents for US tomography, MNPs can be the direct source of ultrasonic waves that can be subsequently used for US imaging 153. It has been demonstrated that such ultrasonic waves can be induced either by the motion of SPIONs exposed to inhomogeneous magnetic fields or as a result of a homogeneous magnetic field via the magnetostriction effect (**Figure 8A**) 153. In MH, MNPs are placed in an alternating magnetic field. In an inhomogeneous magnetic field, the main heating mechanism is related to the translational motion of the MNPs. Hu and He presented an experimental study on magnetoacoustic imaging of SPIONs embedded in biological tissues (**Figure 8C-D**). The US signals induced by the magnetic forces acting on the NPs were measured using a rotating transducer, and the NP distribution was subsequently reconstructed. The results demonstrated the feasibility of obtaining cross-sectional images of MNP targets with reliable dimensional and positional information. Such results suggest the possibility of creating novel tools helpful for US tomographic reconstruction of magnetically labeled tissues 58. The possibility of magnetically generated US in the body (referred to as intracellularly generated US) might be highly beneficial, as typical ultrasonic wave propagation takes place from an outer source into the body and is often limited. Because bones have a higher density and lower compressibility than soft tissues, they create intense ultrasonic wave reflections and refractions. Such distortion and attenuation of transcranial US signals are significant problems in US visualization of the brain, as the lateral resolution is strongly affected and the imaging assessment of the soft tissue structures underlying bones is hampered. Ultrasonic waves generated by MNPs exposed to magnetic fields can also be actively used for US therapy at the cellular level 154. This includes possible solutions for overcoming the BBB such as acoustic induction of bioeffects in cells, manipulation of the permeability of biological membranes 153, and drug release from magnetic liposomes 59.

### 7.2 Magnetic mediators for US thermography

The ability to measure tissue temperature in real-time is a very important aspect of thermal therapies. We have observed increasing interest in the use of US for monitoring temperature changes. As acoustic parameters of tissue change with temperature, correlations between them and the temperature of the medium can be used as a measure of temperature control during hyperthermia. A wide range of US-based methods for temperature monitoring take into account measurements of frequency-dependent attenuation, backscattering coefficients, speed of sound, and thermal expansion. Measurement of the speed of sound is the most popular choice. For instance, images that present the distribution of reconstructed values of speeds of sound can be used for a noninvasive monitoring of the temperature changes. A transmission US scanning system with MNPs as a contrast agent was shown to be able to detect temperature changes with a resolution of <0.5 °C 152. If the temperature distribution of a medium is uniform and homogeneous, the speed of sound measurement can easily contribute to the calculation of temperature. In the case of nonuniform temperature fields, such relations and calculations become complicated 156, 157.

US thermography may be used for monitoring hyperthermia efficacy. Feng *et al.* proposed the integration of MH and thermoacoustic tomography 158. During MH experiments, the magnetic coil induces thermoacoustic signals (acoustic waves induced by thermoelastic expansion due to time-varying heat dissipation) that subsequently can be correlated with the observed temperature increase 159. To obtain temperature images from US signals, plate transducers and post-processing were used (**Figure 8B**). The integration of NP hyperthermia and magnetic field-induced thermoacoustic imaging can potentially contribute to the development of a theranostic platform with imaging and temperature monitoring capabilities superior to conventional imaging modalities 155, 158. Such a method has the potential to revolutionize current cancer treatment by enabling diagnosis and treatment under real-time feedback in one session 155.

Another technique that enables monitoring of temperature rises in tissues is based on estimations of ultrasonic echo displacement. However, when the local temperature rise is not sufficient to be observable during B-mode imaging, special algorithms for calculating the temperature are required. Alternatively, comparison of signals recorded before and after temperature changes can be used for calculation of echo displacements, and thus estimation of sound velocity in tissue and tissue temperature 133. This method is a promising tool for the noninvasive monitoring of temperature fields during various thermal treatments.

Despite the presented applications and noticeable advantages of US tomography, there still exists one main difficulty. A physical model must be established that is able to take into account the full complexity of acoustic phenomena occurring in a relatively small area of interest. This issue partially exists due to the inherent variability between humans. However, the possibility of solving this problem has already been proposed by Kłosowski *et al.* 160*.* The authors showed that neural networks can be used for image reconstruction in US tomography. In light of the remarkable increases in scientific efforts toward improving artificial intelligence algorithms and tailoring them to clinical needs, we can expect further development of US tomography techniques and their introduction as standard procedures. Before US tomography can become a commonly used technique, however, some challenging technical issues due to the complexity of required US probes need to be resolved first. However, we believe that finding a successful solution could contribute to the development of a new technique that is, in some measures, an improved version of US imaging. Subsequently, many theranostic applications could be relatively simply transformed so that they could be used with US tomography.

## 8. Other Applications in Ultrasound Theranostics

Acoustic cluster therapy involves administration of MB or microdroplet clusters together with a chemotherapeutic and ultrasonic sonication of the targeted pathological tissue. When exposed to diagnostic US, the clusters undergo a phase-shift and in consequence produce bubbles. Subsequently, low-frequency US is applied to drive oscillations of the deposited bubbles, which induce locally enhanced extravasation, distribution, and uptake of the co-administered drug. Such multimodal therapy increases the therapeutic efficacy of the anticancer treatment 161. We believe that the use of clusters embedded with MNPs could create additional possibilities such as magnetic targeting. Clusters could then deliver not only the contrast-improving sensitizers to the site of interest but also pharmaceuticals. Reduction of imaging distortions during MRI-guided ultrasonic transcranial thermal ablation can be obtained with the combination of MNPs and US.

Kuhn *et al.* developed trimodal gold-dotted MNPs for MR, CT, and intravascular US imaging 162. The authors demonstrated that such NPs could be successfully used as contrast-enhancing agents to improve imaging performance. The prepared MNPs had pristine surfaces, which were indicated to be favorable for further theranostic applications after functionalization. Zhang *et al.* presented the use of a radiofrequency solenoid vaporization process for enhancing US imaging 163. The authors showed that radiofrequency-induced ablation of tumors was improved by lowering the power of the radiofrequency device and the treatment time. When a water-based SPION suspension replaces degassed water, commonly used for coupling US transducers with the sonicated site, the MR signal induced by the coupling bath itself is beneficially reduced 164. In this application, MNPs were not used as a sonosensitizer but were an integral element for improving US therapy of brain conditions. Although the introduction of a completely novel approach to US-based imaging is rather rare, Huang *et al.* presented an “out-of-the box” idea for using MNPs as mediator 165. US can cause vibration of pre-magnetized MNPs that can subsequently become secondary sources of alternating magnetic signals, which are potentially useful for imaging. This work showed the reverse effect of the MMUS principle presented in Section 6. As it is well known that MNPs in alternating magnetic fields are sources of heat, this method could be improved by adding thermal therapy components. Another example where a new idea was developed by paradigmatically changing traditional imaging techniques is US localization microscopy. By using MBs as enhancers for US imaging and targeting them to the vascular network of the brain, the anatomy of the network can be imaged with a resolution that is impossible to achieve by any other noninvasive imaging technique 166. Development of this technique is highly desirable, as treatment and imaging of fragile brain tissue is still challenging.

## 9. Discussion and Perspective

The essence of theranostic approaches is to use both stimuli and mediators in dual roles. In the case of US theranostics, ultrasonic waves can be used to induce therapeutic effects as well as to image and characterize the host medium (e.g., tissue affected by cancer), while magnetic mediators can improve imaging contrast, induce various therapeutic effects, such as local heating in MH or ROS induction in STD, or deliver therapeutic agents to the site of interest.

By collective analysis of the studies presented in this review, we conclude that some types of sensitizers are more versatile than others. In most cases, complex MNPs can be designed to efficiently mediate theranostics because their fabrication and surface functionalization possibilities are, in theory, infinite. However, MNPs are still mostly used in traditional US diagnostics, where magnetic MBs that are well-characterized and used in clinical routine are much more convenient. In Table 1 we summarized the magnetic mediators for US theranostics presented throughout this review. Their applications and characteristics, such as size, loading method, type of magnetic theranostic agent, and chemical composition, are described.

### 9.1 Challenges of magnetic-based ultrasound theranostic

When focusing on the advantages and future opportunities of ultrasound theranostic approaches, one should also systematically consider their current challenges and limitations. The most critical ones involve the translation and scaleup of reported ultrasound theranostic experimental setups used with cell lines and small animals into setups that would meet clinical standards. Also, acceptable values for intensities, powers, and mediator concentrations for all therapeutic modalities must be established. When we introduce magnetic mediators into the human body, we need to also consider the immune response and inherent risk of toxicity. The toxicity of magnetic mediators varies as it depends on numerous factors, e.g., size, shape, structure, surface modification, concentration, dosage, biodistribution, bioavailability, solubility, immunogenicity, pharmacokinetics. Iron oxide NPs are the most preferred MNP thanks to their good dispersion, and efficient penetration of cell and tissue barriers. They have already been clinically applied as contrast agents in MRI, which proves their efficacy and safety 167. In clinical trials, MNPs did not show any systemic toxicity 16 and are clinically safe for use at a 100-fold greater amount than a dose needed for detection on MRI scanning 90. In detail, iron oxide NPs is safe and non-cytotoxic below 100 mg/mL 168. Therefore it can be confidently considered for using in ultrasound theranostics. Although overcoming the above-mentioned challenges of US theranostics is still very demanding, it seems to be within reach.

### 9.2 Perspective for novel ultrasound theranostic approaches

**Figure 9:**
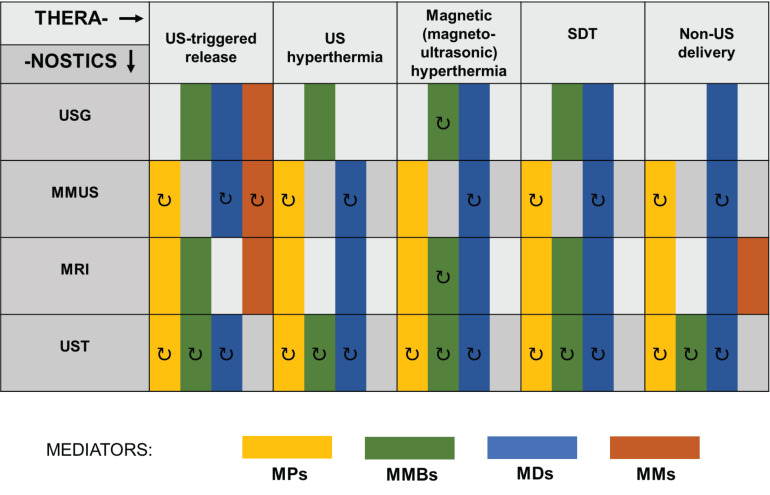
Combinations of imaging and therapeutic techniques involving US and various types of magnetic mediators. MDs, magnetic droplets and liposomes; MMs, magnetic microrobots and other vehicles; MMBs, magnetic microbubbles; MMUS, magnetomotive ultrasound imaging; MPs, magnetic particles; MRI, magnetic resonance imaging; SDT, sonodynamic therapy; US, ultrasound; USG, ultrasonography; UST, ultrasonic tomography. Proposed theranostic perspectives that could emerge in the future are indicated with the symbol **↻**.

In addition to the approaches reviewed in this paper, there still exists a number of other under investigated theranostic applications of US using magnetic mediators. Therefore, we wanted to provide an encouraging roadmap for other scientists on how to make their research directed and open to the possibility of theranostic applications. In **Figure 9**, we schematically presented the existing and possible combinations of imaging and therapeutic techniques involving US and various types of magnetic mediators. Already existing ultrasound theranostic approaches have been presented on Figure 9 as a solid rectangular block. Whereas those still undiscovered have been marked with an additional arrow symbol indicating their possible development. Colors indicate various magnetic mediators that can be used as agents for therapeutic and diagnostic techniques. For instance, in the case of MMUS, ultrasound theranostic applications can be easily developed in the near future, as the MMUS approach inherently utilizes magnetic material (MNP) that can act as a dual-role agent for imaging and therapy. MMUS technique using MNP could be combined with ultrasound hyperthermia for triggered release, or by using magnetic droplets combined with SDT. Such an open area for development is in opposition to the traditional imaging techniques whose basics have been conclusively established and only gradually improved. In addition to currently used magnetic mediators, others can be easily developed and incorporated into ultrasound theranostics. For instance, magnetotactic bacteria proposed as MH mediators 169 and contrast agents for PA imaging 170 can be further easily modified and combined with other mediators, which creates plenty of other possibilities for their use as bioinspired theranostic agents.

Further perspectives for novel theranostic approaches should especially aim to improve the technological aspects of diagnostic and therapeutic apparatus. Their improved efficiency (obtained with safe, low powers) and miniaturization. A promising idea is to use new classes of vehicles, such as nanorobots and nanomotors 33, 34 mentioned in Section 2. What is advantageous, their application is beyond the commonly investigated medical uses of functionalized materials. The intelligence of such mediators combined with their responsiveness to external stimuli (including US) could someday lead to the next paradigm change, as did the development of theranostics.

## 10. Conclusion

Throughout this review, we have presented a variety of approaches that improve the efficiency of therapy and diagnostics, from quite straightforward combinations of US and pristine MNPs to very complex agents whose preparation must be preceded by refinements in design. Some of these have been considered breakthrough technologies due to the uniqueness of their substrate combination or demonstrated high therapeutic efficiency. Most of the presented papers have been published within the last few years, which evidence the significant progress in the area of theranostic materials. However, at the same time, it shows that we have just begun our scientific journey leading towards real clinical applications.

It should be stressed that the vast majority of scientific reports presented experimental results based on simple tissue-mimicking phantoms or animal models that provide an understanding of the ultrasonic/magnetic mechanisms and/or introduce novel combinations of various magnetic mediators that differ in material composition, shape, and size*.* Demonstrating the efficiency of US theranostics in animal models holds promise for their successful transfer into clinical trials. However, the satisfactory efficiency and surprising versatility obtained during laboratory experiments do not necessarily lead to fast and robust establishment of useful clinical procedures (as in the case of MH of cancer). The practical application of MNPs is also a prevalent and ongoing challenge. Ensuring their biocompatibility and biosafety is crucial when considering future clinical trials. Fortunately, this issue seems to now be widely known, and even scientists that are not experts in clinical trials take this into account when designing their theranostic agents. However, it is still an “addition” to scientific investigations rather than the basis of real procedures. The design of biocompatible materials as mediators in US theranostics should not be an aim unto itself. The final goal when creating new agents should be their introduction into working platforms. Even though magnetoacoustic nanomedicine is still in its infancy, the prospective clinical potential makes it worth extensively exploring 10, while keeping in mind that the main goal is to create efficient medical platforms not scientific papers.

To summarize, the numerous studies presented and discussed in this review show the existing potential for various magneto-ultrasonic diagnostics and therapies. The greatest numbers of magneto-ultrasonic therapy applications have been proposed for drug delivery and release, and the additional presence of magnetic mediators can noticeably improve magnetic- and ultrasonic-based imaging modalities. Moreover, *in vitro* and *in vivo* studies have confirmed the possibility of creating multimodal imaging and therapy approaches that could improve or even replace traditional single mode techniques. The continuous improvement and development of theranostic materials, including MNPs, contributes to the advancement of diagnostic and therapeutic methods. The use of sensitizers allows for lowering of acoustic/magnetic/electromagnetic intensities and therefore creates possibilities for emerging devices, physical models, and computing algorithms. Though many questions are still open and various issues still need to be resolved, one may state without a doubt that theranostics has presented significant scientific progress and gives hope for achieving more effective strategies for cancer treatment. Although many US-based theranostic possibilities have been presented in this review, there still exists undeniable potential for further approaches.

## Figures and Tables

**Table 1:** Overview of magnetic agents proposed for US theranostic applications.

Type of theranostic magnetic agents	Materials	The way of agent loading	Size of agents	US theranostic application	Reference
droplets	Chitosan- deoxycholic acid nanoparticles (shell), perfluoropentane droplets and iron oxide particles (core)	siRNA electrostatically bounded to particles; MPs inside the droplet	257.6±10.9 nm	US-imaged US-induced delivery & Magnetic localization	25
	iron oxide nanoparticles, doxorubicin (drug), antibody molecules, perfluoropentane (gas), phospholipid layer	lipid shell-coated droplet with antibodies with encapsulated magnetic particles and doxorubicin	1.49±0.24 µm	MR-guided US-induced delivery	62
spheres	polystyrene template, silica hollow nanoparticles modified with Gd-DTP acid and c(RGD) peptide	peptides attached to the sphere surface	100-400 nm	US and MR-imaged magnetic targeting	26
	polyglutamic acid (PGA)-stabilized iron oxide porous nanoparticles, polymethacrylic acid (PMMA), yolk-shell doxorubicin (drug), perfluorohexane (gas)	magnetic particles coated with PGA covered by PMMA layer and the shell of yolk with doxorubicin and perfluorohexane loaded inside	~840 nm	US and MR-imaged US-induced delivery	53
	iron oxide nanoparticles, perfluorohexane (gas)	perfluorohexane encapsulated inside porous magnetic spheres	845±65.8 nm	US and MR-imaged magnetically-induced delivery	61
bubbles	octafluoropropane (gas) phospholipids with protamine conjugated with PEG (coating), iron oxide nanoparticles, heparin (shell)	heparin-functionalized iron oxide nanoparticles attached to the surface of microbubble's lipid shell	3.1±1.4 μm	US-imaged US-induced delivery & Magnetic targeting	29
	perfluoropropane (gas core), nucleic acid, iron oxide nanoparticles, metafectene/DOPE soybean oil (lipid shell)	magnetic nanoparticles loaded in the bubble shell with plasmid DNA and siRNA	3±2.3 μm	US-imaged US-induced delivery & Magnetic targeting	31
	iron oxide nanoparticles, poly(DL -lactide) (PLA), nitrogen (gas)	magnetic particles attached to polymer shell	3-5 µm	MR-imaged US-induced delivery	63
	iron oxide nanoparticles, PLGA-PEG-folate polymer, doxorubicin (drug)	magnetic particles and doxorubicin encapsulated into polymer shell	208.4±12.58 nm	MR and US-imaged US-induced delivery	64
	iron oxide nanoparticles, perfluoropentane (gas), Pluronic F127 (drug), polyacrylic acid polymers	perfluoropentane encapsulated in a polymer shell stabilized with magnetic particles	180-230 nm	MR and US-imaged US-induced delivery & Magnetic targeting	65
	iron oxide nanoparticles, poly( n-butyl-cyanoacrylate), fluorescein isothiocyanate (FITC)-dextran (drug model), air	magnetic particles embedded in the shell	1-5 µm	MR-imaged US-induced blood-brain barrier permeation & Magnetic targeting	54
	iron oxide nanoparticles, perfluoropentane (gas), fingolimod (drug)	magnetic particles and drug molecules and gas as a core coated with RGD-modified liposomal shell	160-200 nm	MR and US-imaged US-induced delivery & Magnetic targeting	56
	iron oxide nanoparticles, doxorubicin, perfluoropropane (gas), lipid shell	doxorubicin-conjugated magnetic particles embedded in lipid shell around gas core	3.1±0.2 µm	MR-imaged US-induced blood-brain barrier permeation & Magnetic targeting	47
	iron oxide nanoparticles, perfluoropentane (gas), dextran	magnetic particles-covered dextran layer around gas core	349.2±18.2 nm	MR and US-imaged magnetic targeting	48
	iron oxide nanoparticles, oleic acid, perfluoropentane (gas), silane-based layer	oleic acid-coated magnetic particles embedded in the silane-based shell	200-2000 nm	MR-imaged US-induced blood-brain barrier permeation & Magnetic targeting	49
	iron oxide nanoparticles, herceptin (anticancer drug), paclitaxel (anticancer drug), octafluoropropane (gas), PLGA polymer	herceptin-decorated magnetic nanoparticles with paclitaxel embedded PLGA later of bubbles with gas core	277.9-309.9 nm	MR, US and PA-imaged US-induced delivery	44
	iron oxide nanoparticles, poly(lactic-co-glycolic acid) (PLGA) polymer, doxorubicin, perfluorocarbon (gas)	magnetic particles and doxorubicin co-encapsulated into PLGA layer around gas core	868 nm±68.73 nm	MR and US-imaged US-induced delivery	45
capsules	tannic acid and polyvinylpyrrolidone layer, iron oxide nanoparticles modified with tannic acid, doxorubicin (drug)	iron oxide nanoparticles embedded into the layer; doxorubicin loaded to the capsules	~3 µm	MR-imaged US-induced delivery	40
	polystyrene-PAA polymer, PEG (coating), perfluorooctyl-bromide nanoparticles particles, iron oxide nanoparticles	PEGylated magnetite/perfluorooctyl-bromide-loaded encapsulated perfluorocarbon and magnetic particles inside the layer of PS-PAA and PEG coating	~175 nm	MR and US-imaged US-induced thermal ablation	82
nanoparticles	iron oxide nanoparticles, mesoporous silica nanoparticles, CTAB	iron oxide core, mesoporous silica shell, dibenzo-crown ethers coupled onto shell, doxorubicin loaded into pores of particles	~200 nm	MR-imaged US-induced delivery	41
	iron oxide nanoparticles, polyaniline-co-sodium, carmustine (anticancer drug)	carmustine immobilized on the surface of magnetic particles coated with polyaniline-co-sodium	10-20 nm	MR-imaged US-induced blood-brain barrier permeation & Magnetic targeting	46
	PEG (coating), iron oxide nanoparticles, antibodies	PEGylated magnetic particles decorated with antibodies	~45.7 nm	MR-imaged US-induced thermal ablation	83
	mesoporous silica, Rose Bengal (sonosensitizers), PEG (coating), iron oxide nanoparticles, graphene oxide nanosheet	porous silica nanoparticles grown on graphene nanosheet and capped with Rose Bengal-PEG conjugated magnetic particles	~60 nm	MR-imaged US-induced thermal ablation	84
	titanium oxide nanoparticles, gadolinium doxorubicin, folic acid	drug molecules linked with titanium oxide -gadolinium particles covered with folic acid	~100 nm	MR-imaged SDT	110
	mesoporous silica nanoparticles, protoporphyrin (sonosensitizer), manganese	protoporphyrin with chelated manganese ions loaded into mesoporous silica nanoparticles	~50 nm	MR-imaged SDT	115
	PEG-grafted phosphorylated serine, bismuth ferrite nanoparticles	bismuth ferrite particles modified by PEG-grafted phosphorylated serine	~48.7 nm	MR-imaged SDT	116
	manganese oxide nanocrystals, protoporphyrin (sonosensitizer), holo-transferrin	manganese oxide crystals grown into holo-transferrin and protoporphyrin as sonosensitizer introduced into the holo-transferrin	~30 nm	MR-imaged SDT	118
	hollow iron oxide nanoparticles, hematoporphyrin (sonosensitizer), polydopamine	polydopamine and PEG coated the surface of magnetic particles loaded with hematoporphyrin	526.24 ± 48.89 nm	PA-imaged MH and SDT	122
	zinc, iron oxide nanoparticles	zinc-substituted magnetite particles without coating	12.4 ±2 nm	MMUS-imaged MH	146
	iron oxide nanoparticles, oleic acid	magnetic particles coated with oleic acid	6.1 ± 1.5 nm	US-imaged MH	155
liposomes	iron oxide nanoparticles, phospholipides , combretastatin A-4 Phosphate (vascular disrupting agent)	drug encapsulated in the aqueous part of the liposome with magnetic particles	209 ± 56 nm	MR-imaged US-induced delivery & Magnetic targeting	43
	iron oxide nanoparticles, anethole dithiolethione, hydrogen sulfide (gas)	anethole dithiolethione and hydrogen sulfide doped in the lipid layer, magnetic particles encapsulated inside	211.1 ± 4.64 nm	MR and US-imaged US-induced thermal ablation	60
	sinoporphyrin sodium (sonosensitizer), manganese, lipids	paramagnetic manganese anchored into sinoporphyrin sodium molecules and encalsupaled into lipid shell	85.15 ± 2.14 nm	MR-imaged SDT	114
nanomotors	bacterial strains of E. coli and S. aureus, gold and nickel metallic nanowires	bacteria captured by bioreceptors covering the nanomotor gold surface drug conjugated to the segment by electrostatic forces and released by pH change	0.25 μm (diameter), 1.8 μm (length)	MR-imaged US-induced targeting	32
hairbots	hair shafts, iron oxide nanoparticles, doxorubicin (drug)	hairbots coated with PEGylated iron oxide nanoparticles; doxorubicin loaded into hairbots through precipitation and hydrophobic interactions	10 μm (thickness), 60-80 μm (lateral dimensions)	US-imaged US-induced delivery & Magnetic targeting	33
microrobots	NdFeB magnetic microparticles, doxorubicin (drug)	dispersion of magnetic nanoparticles as an ink injected into chamber; doxorubicin attached to robot's surface	several mm	US-induced delivery & Magnetic targeting	34
nanodots	iron, titanium dioxide, PEG (coating)	iron-doped titanium oxide modified with PEG	2.49-2.73 nm	MR-imaged SDT	111

## References

[1] Lim E-K, Kim T, Paik S, Haam S, Huh Y-M, Lee K (2015). Nanomaterials for theranostics: recent advances and future challenges. Chem Rev.

[2] Chen J, Ratnayaka S, Alford A, Kozlovskaya V, Liu F, Xue B (2017). Theranostic multilayer capsules for ultrasound imaging and guided drug delivery. ACS Nano.

[3] Miller DL, Smith NB, Bailey MR, Czarnota GJ, Hynynen K, Makin IRS (2012). Overview of therapeutic ultrasound applications and safety considerations. J Ultrasound Med.

[4] Miller DB, O'Callaghan JP (2017). New horizons for focused ultrasound (FUS)-therapeutic applications in neurodegenerative diseases. Metabolism.

[5] Zhou L-Q, Li P, Cui X-W, Dietrich CF (2020). Ultrasound nanotheranostics in fighting cancer: advances and prospects. Cancer Lett.

[6] Ellens N, Hynynen K (2015). High-intensity focused ultrasound for medical therapy. In: Gallego-Juárez JA, Ed. Power Ultrasonics, Cambridge: Woodhead Publishing.

[7] Qian X, Han X, Chen Y (2017). Insights into the unique functionality of inorganic micro/nanoparticles for versatile ultrasound theranostics. Biomaterials.

[8] Opieliński KJ, Pruchnicki P, Szymanowski P, Szepieniec WK, Szweda H, Świś E (2018). Multimodal ultrasound computer-assisted tomography: an approach to the recognition of breast lesions. Comput Med Imaging Graph.

[9] Frazier N, Ghandehari H (2015). Hyperthermia approaches for enhanced delivery of nanomedicines to solid tumors. Biotechnol Bioeng.

[10] Devarakonda SB, Myers MR, Lanier M, Dumoulin C, Banerjee RK (2017). Assessment of gold nanoparticle-mediated-enhanced hyperthermia using MR-guided high-intensity focused ultrasound ablation procedure. Nano Lett.

[11] Beik J, Abed Z, Ghadimi-Daresajini A, Nourbakhsh M, Shakeri-Zadeh A, Ghasemi MS (2016). Measurements of nanoparticle-enhanced heating from 1 MHz ultrasound in solution and in mice bearing CT26 colon tumors. J Therm Biol.

[12] Duan L, Yang L, Jin J, Yang F, Liu D, Hu K (2020). Micro/nano-bubble-assisted ultrasound to enhance the EPR effect and potential theranostic applications. Theranostics.

[13] Frinking P, Segers T, Luan Y, Tranquart F (2020). Three decades of ultrasound contrast agents: a review of the past, present and future improvements. Ultrasound Med Biol.

[14] Abenojar EC, Bederman I, de Leon AC, Zhu J, Hadley J, Kolios MC (2020). Theoretical and experimental gas volume quantification of micro- and nanobubble ultrasound contrast agents. Pharmaceutics.

[15] Fan Z, Fu PP, Yu H, Ray PC (2014). Theranostic nanomedicine for cancer detection and treatment. J Food Drug Anal.

[16] Thiesen B, Jordan A (2008). Clinical applications of magnetic nanoparticles for hyperthermia. Int J Hyperth.

[17] Deatsch AE, Evans BA (2014). Heating efficiency in magnetic nanoparticle hyperthermia. J Magn Magn Mater.

[18] Arranja AG, Pathak V, Lammers T, Shi Y (2017). Tumor-targeted nanomedicines for cancer theranostics. Pharmacol Res.

[19] Jani P, Subramanian S, Korde A, Rathod L, Sawant KK (2020). Theranostic nanocarriers in cancer: dual capabilities on a single platform. In: Thangadurai D, Sangeetha J, Prasad R, Eds Functional Bionanomaterials New York: Springer.

[20] Wang W, Huang Z, Huang Y, Pan X, Wu C (2020). Updates on the applications of iron-based nanoplatforms in tumor theranostics. Int J Pharm.

[21] Saravanakumar K, Hu X, Ali DM, Wang M-H (2019). Emerging strategies in stimuli-responsive nanocarriers as the drug delivery system for enhanced cancer therapy. Curr Pharm Des.

[22] Chen L, Wu Y, Wu H, Li J, Xie J, Zang F (2019). Magnetic targeting combined with active targeting of dual-ligand iron oxide nanoprobes to promote the penetration depth in tumors for effective magnetic resonance imaging and hyperthermia. Acta Biomater.

[23] Zhu L, Zhou Z, Mao H, Yang L (2017). Magnetic nanoparticles for precision oncology: theranostic magnetic iron oxide nanoparticles for image-guided and targeted cancer therapy. Nanomedicine.

[24] Liu D, Yang F, Xiong F, Gu N (2016). The smart drug delivery system and its clinical potential. Theranostics.

[25] Lee JY, Crake C, Teo B, Carugo D, de Saint Victor M, Seth A (2017). Ultrasound-enhanced siRNA delivery using magnetic nanoparticle-loaded chitosan-deoxycholic acid nanodroplets. Adv Healthc Mater.

[26] An L, Hu H, Du J, Wei J, Wang L, Yang H (2014). Paramagnetic hollow silica nanospheres for *in vivo* targeted ultrasound and magnetic resonance imaging. Biomaterials.

[27] Li X, Xia S, Zhou W, Ji R, Zhan W (2019). Targeted Fe-doped silica nanoparticles as a novel ultrasound-magnetic resonance dual-mode imaging contrast agent for HER2-positive breast cancer. Int J Nanomed.

[28] Ji R, Li X, Zhou C, Tian Q, Li C, Xia S (2018). Identifying macrophage enrichment in atherosclerotic plaques by targeting dual-modal US imaging/MRI based on biodegradable Fe-doped hollow silica nanospheres conjugated with anti-CD68 antibody. Nanoscale.

[29] Chertok B, Langer R (2018). Circulating magnetic microbubbles for localized real-time control of drug delivery by ultrasonography-guided magnetic targeting and ultrasound. Theranostics.

[30] Holzbach T, Vlaskou D, Neshkova I, Konerding MA, Wörtler K, Mykhaylyk O (2010). Non-viral VEGF165 gene therapy - magnetofection of acoustically active magnetic lipospheres ('magnetobubbles') increases tissue survival in an oversized skin flap model. J Cell Mol Med.

[31] Vlaskou D, Mykhaylyk O, Krötz F, Hellwig N, Renner R, Schillinger U (2010). Magnetic and acoustically active lipospheres for magnetically targeted nucleic acid delivery. Adv Funct Mater.

[32] Garcia-Gradilla V, Orozco J, Sattayasamitsathit S, Soto F, Kuralay F, Pourazary A (2013). Functionalized ultrasound-propelled magnetically guided nanomotors: toward practical biomedical applications. ACS Nano.

[33] Singh AV, Dad Ansari MH, Dayan CB, Giltinan J, Wang S, Yu Y (2019). Multifunctional magnetic hairbot for untethered osteogenesis, ultrasound contrast imaging and drug delivery. Biomaterials.

[34] Darmawan BA, Lee SB, Nguyen VD, Go G, Nguyen KT, Lee H-S (2020). Self-folded microrobot for active drug delivery and rapid ultrasound-triggered drug release. Sens Actuators B Chem.

[35] Kariminia S, Shamsipur A, Shamsipur M (2016). Analytical characteristics and application of novel chitosan coated magnetic nanoparticles as an efficient drug delivery system for ciprofloxacin. Enhanced drug release kinetics by low-frequency ultrasounds. J Pharm Biomed Anal.

[36] Sengupta S, Khatua C, Jana A, Balla VK (2018). Use of ultrasound with magnetic field for enhanced *in vitro* drug delivery in colon cancer treatment. J Mater Res.

[37] Sengupta S, Khatua C, Pal A, Bodhak S, Balla VK (2020). Influence of ultrasound and magnetic field treatment time on carcinoma cell inhibition with drug carriers: an *in vitro* study. Ultrasound Med Biol.

[38] Hynynen K (2010). MRI-guided focused ultrasound treatments. Ultrasonics.

[39] Thanou M, Gedroyc W (2013). MRI-guided focused ultrasound as a new method of drug delivery. J Drug Deliv.

[40] Alford A, Rich M, Kozlovskaya V, Chen J, Sherwood J, Bolding M (2018). Ultrasound-triggered delivery of anticancer therapeutics from MRI-visible multilayer microcapsules. Adv Ther.

[41] Lee S-F, Zhu X-M, Wang Y-XJ, Xuan S-H, You Q, Chan W-H (2013). Ultrasound, pH, and magnetically responsive crown-ether-coated core/shell nanoparticles as drug encapsulation and release systems. ACS Appl Mater Interfaces.

[42] Shakeri-Zadeh A, Khoee S, Shiran M-B, Sharifi AM, Khoei S (2015). Synergistic effects of magnetic drug targeting using a newly developed nanocapsule and tumor irradiation by ultrasound on CT26 tumors in BALB/c mice. J Mater Chem B.

[43] Thébault CJ, Ramniceanu G, Boumati S, Michel A, Seguin J, Larrat B (2020). Theranostic MRI liposomes for magnetic targeting and ultrasound triggered release of the antivascular CA4P. J Control Release.

[44] Song W, Luo Y, Zhao Y, Liu X, Zhao J, Luo J (2017). Magnetic nanobubbles with potential for targeted drug delivery and trimodal imaging in breast cancer: an *in vitro* study. Nanomedicine.

[45] Niu C, Wang Z, Lu G, Krupka TM, Sun Y, You Y (2013). Doxorubicin loaded superparamagnetic PLGA-iron oxide multifunctional microbubbles for dual-mode US/MR imaging and therapy of metastasis in lymph nodes. Biomaterials.

[46] Chen P-Y, Liu H-L, Hua M-Y, Yang H-W, Huang C-Y, Chu P-C (2010). Novel magnetic/ultrasound focusing system enhances nanoparticle drug delivery for glioma treatment. Neuro Oncol.

[47] Fan C-H, Cheng Y-H, Ting C-Y, Ho Y-J, Hsu P-H, Liu H-L (2016). Ultrasound/magnetic targeting with SPIO-DOX-microbubble complex for image-guided drug delivery in brain tumors. Theranostics.

[48] Ficiarà E, Ansari SA, Argenziano M, Cangemi L, Monge C, Cavalli R (2020). Beyond oncological hyperthermia: physically drivable magnetic nanobubbles as novel multipurpose theranostic carriers in the central nervous system. Molecules.

[49] Huang H-Y, Liu H-L, Hsu P-H, Chiang C-S, Tsai C-H, Chi H-S (2015). A multitheragnostic nanobubble system to induce blood-brain barrier disruption with magnetically guided focused ultrasound. Adv Mater.

[50] Carpentier A, Canney M, Vignot A, Reina V, Beccaria K, Horodyckid C (2016). Clinical trial of blood-brain barrier disruption by pulsed ultrasound. Sci Transl Med.

[51] Gao Y, Chan CU, Gu Q, Lin X, Zhang W, Yeo DCL (2016). Controlled nanoparticle release from stable magnetic microbubble oscillations. NPG Asia Mater.

[52] Dwivedi P, Kiran S, Han S, Dwivedi M, Khatik R, Fan R (2020). Magnetic targeting and ultrasound activation of liposome-microbubble conjugate for enhanced delivery of anticancer therapies. ACS Appl Mater Interfaces.

[53] Yang P, Luo X, Wang S, Wang F, Tang C, Wang C (2017). Biodegradable yolk-shell microspheres for ultrasound/MR dual-modality imaging and controlled drug delivery. Colloids Surf B.

[54] Lammers T, Koczera P, Fokong S, Gremse F, Ehling J, Vogt M (2015). Theranostic USPIO-loaded microbubbles for mediating and monitoring blood-brain barrier permeation. Adv Funct Mater.

[55] Yildirim A, Blum NT, Goodwin AP (2019). Colloids, nanoparticles, and materials for imaging, delivery, ablation, and theranostics by focused ultrasound (FUS). Theranostics.

[56] Guo X-M, Chen J-L, Zeng B-H, Lai J-C, Lin C-Y, Lai M-Y (2020). Ultrasound-mediated delivery of RGD-conjugated nanobubbles loaded with fingolimod and superparamagnetic iron oxide nanoparticles: targeting hepatocellular carcinoma and enhancing magnetic resonance imaging. RSC Advances.

[57] Owen J, Crake C, Lee JY, Carugo D, Beguin E, Khrapitchev AA (2018). A versatile method for the preparation of particle-loaded microbubbles for multimodality imaging and targeted drug delivery. Drug Deliv Transl Res.

[58] Hu G, He B (2012). Magnetoacoustic imaging of magnetic iron oxide nanoparticles embedded in biological tissues with microsecond magnetic stimulation. Appl Phys Lett.

[59] Podaru G, Ogden S, Baxter A, Shrestha T, Ren S, Thapa P (2014). Pulsed magnetic field induced fast drug release from magneto liposomes via ultrasound generation. J Phys Chem B.

[60] Liu Y, Yang F, Yuan C, Li M, Wang T, Chen B (2017). Magnetic nanoliposomes as *in situ* microbubble bombers for multimodality image-guided cancer theranostics. ACS Nano.

[61] Wang R, Zhou Y, Zhang P, Chen Y, Gao W, Xu J (2017). Phase-transitional Fe3O4 perfluorohexane microspheres for magnetic droplet vaporization. Theranostics.

[62] Wang C-H, Kang S-T, Yeh C-K (2013). Superparamagnetic iron oxide and drug complex-embedded acoustic droplets for ultrasound targeted theranosis. Biomaterials.

[63] Yang F, Zhang M, He W, Chen P, Cai X, Yang L (2011). Controlled release of Fe3O4 nanoparticles in encapsulated microbubbles to tumor cells via sonoporation and associated cellular bioeffects. Small.

[64] Jin Z, Chang J, Dou P, Jin S, Jiao M, Tang H (2020). Tumor targeted multifunctional magnetic nanobubbles for MR/US dual imaging and focused ultrasound triggered drug delivery. Front Bioeng Biotechnol.

[65] Huang H-Y, Hu S-H, Hung S-Y, Chiang C-S, Liu H-L, Chiu T-L (2013). SPIO nanoparticle-stabilized PAA-F127 thermosensitive nanobubbles with MR/US dual-modality imaging and HIFU-triggered drug release for magnetically guided *in vivo* tumor therapy. J Control Release.

[66] Tang H, Guo Y, Peng L, Fang H, Wang Z, Zheng Y (2018). *In vivo* Targeted, Responsive, and Synergistic Cancer Nanotheranostics by Magnetic Resonance Imaging-Guided Synergistic High-Intensity Focused Ultrasound Ablation and Chemotherapy. ACS Appl Mater Interfaces.

[67] Phung DC, Nguyen HT, Phuong Tran TT, Jin SG, Yong CS, Truong DH (2019). Combined hyperthermia and chemotherapy as a synergistic anticancer treatment. J Pharm Investig.

[68] Arthur RM, Straube W, Trobaugh J, Moros E (2005). Non-invasive estimation of hyperthermia temperatures with ultrasound. Int J Hyperth.

[69] Silvio D, Rudolf H (2014). Magnetic particle hyperthermia—a promising tumour therapy?. Nanotechnology.

[70] Chen C-W, Lee P-H, Chan Y-C, Hsiao M, Chen C-H, Wu PC (2015). Plasmon-induced hyperthermia: hybrid upconversion NaYF 4: Yb/Er and gold nanomaterials for oral cancer photothermal therapy. J Mater Chem B.

[71] Kim KS, Kim J, Lee JY, Matsuda S, Hideshima S, Mori Y (2016). Stimuli-responsive magnetic nanoparticles for tumor-targeted bimodal imaging and photodynamic/hyperthermia combination therapy. Nanoscale.

[72] Piper RJ, Hughes MA, Moran CM, Kandasamy J (2016). Focused ultrasound as a non-invasive intervention for neurological disease: a review. Br J Neurosurg.

[73] Sviridov A, Tamarov K, Fesenko I, Xu W, Andreev V, Timoshenko V (2019). Cavitation induced by Janus-like mesoporous silicon nanoparticles enhances ultrasound hyperthermia. Front Chem.

[74] Beik J, Abed Z, Shakeri-Zadeh A, Nourbakhsh M, Shiran MB (2016). Evaluation of the sonosensitizing properties of nano-graphene oxide in comparison with iron oxide and gold nanoparticles. Phys E: Low-Dimens Syst and Nanostructures.

[75] Kaczmarek K, Hornowski T, Kubovčíková M, Timko M, Koralewski M, Józefczak A (2018). Heating induced by therapeutic ultrasound in the presence of magnetic nanoparticles. ACS Appl Mater Interfaces.

[76] Holmes M, Povey M (2017). Ultrasonic particle sizing in emulsions. Ultrasound Food Proc Recent Adv.

[77] Kaczmarek K, Bielas R, Siluk M, Hornowski T, Józefczak A (2020). Comparison of magnetic and non-magnetic nanoparticles as sonosensitizers in ultrasonic hyperthermia. Acta Phys Pol A.

[78] Józefczak A, Kaczmarek K, Kubovčíková M, Rozynek Z, Hornowski T (2017). The effect of magnetic nanoparticles on the acoustic properties of tissue-mimicking agar-gel phantoms. J Magn Magn Mater.

[79] Kaczmarek K, Mrówczyński R, Hornowski T, Bielas R, Józefczak A (2019). The effect of tissue-mimicking phantom compressibility on magnetic hyperthermia. Nanomaterials.

[80] Devarakonda SB, Myers MR, Giridhar D, Dibaji SAR, Banerjee RK (2017). Enhanced thermal effect using magnetic nano-particles during high-intensity focused ultrasound. PLOS ONE.

[81] Kaczmarek K, Hornowski T, Dobosz B, Józefczak A (2018). Influence of magnetic nanoparticles on the focused ultrasound hyperthermia. Materials.

[82] Niu D, Wang X, Li Y, Zheng Y, Li F, Chen H (2013). Facile synthesis of magnetite/perfluorocarbon co-loaded organic/inorganic hybrid vesicles for dual-modality ultrasound/magnetic resonance imaging and imaging-guided high-intensity focused ultrasound ablation. Adv Mater.

[83] Wang Z, Qiao R, Tang N, Lu Z, Wang H, Zhang Z (2017). Active targeting theranostic iron oxide nanoparticles for MRI and magnetic resonance-guided focused ultrasound ablation of lung cancer. Biomaterials.

[84] Chen Y-W, Liu T-Y, Chang P-H, Hsu P-H, Liu H-L, Lin H-C (2016). A theranostic nrGO@MSN-ION nanocarrier developed to enhance the combination effect of sonodynamic therapy and ultrasound hyperthermia for treating tumor. Nanoscale.

[85] Yildirimer L, Thanh NTK, Loizidou M, Seifalian AM (2011). Toxicology and clinical potential of nanoparticles. Nano Today.

[86] Kucharczyk K, Kaczmarek K, Jozefczak A, Slachcinski M, Mackiewicz A, Dams-Kozlowska H (2021). Hyperthermia treatment of cancer cells by the application of targeted silk/iron oxide composite spheres. Mater Sci Eng C.

[87] Lahonian M (2013). Diffusion of magnetic nanoparticles within a biological tissue during magnetic fluid hyperthermia. In: Huilgol N, Nanavati B, Eds Hyperthermia, London: INTECH.

[88] Engelmann U, Buhl EM, Baumann M, Schmitz-Rode T, Slabu I (2017). Agglomeration of magnetic nanoparticles and its effects on magnetic hyperthermia. Curr Direct Biomed Eng.

[89] Guo X, Li W, Luo L, Wang Z, Li Q, Kong F (2017). External magnetic field-enhanced chemo-photothermal combination tumor therapy via iron oxide nanoparticles. ACS Appl Mater Interfaces.

[90] Vakili-Ghartavol R, Momtazi-Borojeni AA, Vakili-Ghartavol Z, Aiyelabegan HT, Jaafari MR, Rezayat SM (2020). Toxicity assessment of superparamagnetic iron oxide nanoparticles in different tissues. Artif Cells Nanomed Biotechnol.

[91] Sakellari D, Brintakis K, Kostopoulou A, Myrovali E, Simeonidis K, Lappas A (2016). Ferrimagnetic nanocrystal assemblies as versatile magnetic particle hyperthermia mediators. Mater Sci Eng C.

[92] Avolio M, Guerrini A, Brero F, Innocenti C, Sangregorio C, Cobianchi M (2019). In-gel study of the effect of magnetic nanoparticles immobilization on their heating efficiency for application in Magnetic Fluid Hyperthermia. J Magn Magn Mater.

[93] Engelmann UM, Seifert J, Mues B, Roitsch S, Ménager C, Schmidt AM (2019). Heating efficiency of magnetic nanoparticles decreases with gradual immobilization in hydrogels. J Magn Magn Mater.

[94] Kaczmarek K, Hornowski T, Antal I, Rajnak M, Timko M, Józefczak A (2020). Sono-magnetic heating in tumor phantom. J Magn Magn Mater.

[95] Kaczmarek K, Hornowski T, Antal I, Timko M, Józefczak A (2019). Magneto-ultrasonic heating with nanoparticles. J Magn Magn Mater.

[96] Yan H, Shang W, Sun X, Zhao L, Wang J, Xiong Z (2018). “All-in-one” nanoparticles for trimodality imaging-guided intracellular photo-magnetic hyperthermia therapy under intravenous administration. Adv Funct Mater.

[97] Espinosa A, Di Corato R, Kolosnjaj-Tabi J, Flaud P, Pellegrino T, Wilhelm C (2016). Duality of iron oxide nanoparticles in cancer therapy: amplification of heating efficiency by magnetic hyperthermia and photothermal bimodal treatment. ACS Nano.

[98] Chen H, Di Y, Chen D, Madrid K, Zhang M, Tian C (2015). Combined chemo-and photo-thermal therapy delivered by multifunctional theranostic gold nanorod-loaded microcapsules. Nanoscale.

[99] Mendes R, Pedrosa P, Lima JC, Fernandes AR, Baptista PV (2017). Photothermal enhancement of chemotherapy in breast cancer by visible irradiation of gold nanoparticles. Sci Rep.

[100] Ahmad Reza Dibaji S, Al-Rjoub MF, Myers MR, Banerjee RK (2013). Enhanced heat transfer and thermal dose using magnetic nanoparticles during HIFU thermal ablation—an in-vitro study. J Nanotechnol Eng Med.

[101] Shen S, Huang D, Cao J, Chen Y, Zhang X, Guo S (2019). Magnetic liposomes for light-sensitive drug delivery and combined photothermal-chemotherapy of tumors. J Mater Chem B.

[102] Mérida F, Rinaldi C, Juan EJ, Torres-Lugo M (2020). *In vitro* ultrasonic potentiation of 2-phenylethynesulfonamide/magnetic fluid hyperthermia combination treatments for ovarian cancer. Int J Nanomed.

[103] Yumita N, Nishigaki R, Umemura K, Umemura Si (1989). Hematoporphyrin as a sensitizer of cell-damaging effect of ultrasound. Jpn J Cancer Res.

[104] Hayyan M, Hashim MA, AlNashef IM (2016). Superoxide ion: generation and chemical implications. Chem Rev.

[105] Yan P, Liu L-H, Wang P (2020). Sonodynamic therapy (SDT) for cancer treatment: advanced sensitizers by ultrasound activation to injury tumor. ACS Appl Bio Mater.

[106] Jin H, Zhong X, Wang Z, Huang X, Ye H, Ma S (2011). Sonodynamic effects of hematoporphyrin monomethyl ether on CNE-2 cells detected by atomic force microscopy. J Cell Biochem.

[107] Wang L, Niu M, Zheng C, Zhao H, Niu X, Li L (2018). A core-shell nanoplatform for synergistic enhanced sonodynamic therapy of hypoxic tumor via cascaded strategy. Adv Healthc Mater.

[108] Xu M, Zhou L, Zheng L, Zhou Q, Liu K, Mao Y (2021). Sonodynamic therapy-derived multimodal synergistic cancer therapy. Cancer Lett.

[109] Shen S, Guo X, Wu L, Wang M, Wang X, Kong F (2014). Dual-core@shell-structured Fe3O4-NaYF4@TiO2 nanocomposites as a magnetic targeting drug carrier for bioimaging and combined chemo-sonodynamic therapy. J Mater Chem B.

[110] Yuan P, Song D (2018). MRI tracing non-invasive TiO2-based nanoparticles activated by ultrasound for multi-mechanism therapy of prostatic cancer. Nanotechnology.

[111] Bai S, Yang N, Wang X, Gong F, Dong Z, Gong Y (2020). Ultrasmall iron-doped titanium oxide nanodots for enhanced sonodynamic and chemodynamic cancer therapy. ACS Nano.

[112] Zhu H-m, He Y, Huang S-s, Tian J-j, Wang L-s, Hao J-d (2020). Chlorin e6-loaded sonosensitive magnetic nanoliposomes conjugated with the magnetic field for enhancing anti-tumor effect of sonodynamic therapy. Pharm Dev Technol.

[113] Wang X, Niu D, Li P, Wu Q, Bo X, Liu B (2015). Dual-enzyme-loaded multifunctional hybrid nanogel system for pathological responsive ultrasound imaging and T2-weighted magnetic resonance imaging. ACS Nano.

[114] Liu H, Zhou M, Sheng Z, Chen Y, Yeh C-K, Chen W (2018). Theranostic nanosensitizers for highly efficient MR/fluorescence imaging-guided sonodynamic therapy of gliomas. J Cell Mol Med.

[115] Huang P, Qian X, Chen Y, Yu L, Lin H, Wang L (2017). Metalloporphyrin-encapsulated biodegradable nanosystems for highly efficient magnetic resonance imaging-guided sonodynamic cancer therapy. J Am Chem Soc.

[116] Feng L, Gai S, He F, Yang P, Zhao Y (2020). Multifunctional bismuth ferrite nanocatalysts with optical and magnetic functions for ultrasound-enhanced tumor theranostics. ACS Nano.

[117] Gorgizadeh M, Azarpira N, Lotfi M, Daneshvar F, Salehi F, Sattarahmady N (2019). Sonodynamic cancer therapy by a nickel ferrite/carbon nanocomposite on melanoma tumor: *In vitro* and *in vivo* studies. Photodiagnosis Photodyn Ther.

[118] Liang K, Li Z, Luo Y, Zhang Q, Yin F, Xu L (2020). Intelligent nanocomposites with intrinsic blood-brain-barrier crossing ability designed for highly specific MR imaging and sonodynamic therapy of glioblastoma. Small.

[119] Gorgizadeh M, Behzadpour N, Salehi F, Daneshvar F, Vais RD, Nazari-Vanani R (2020). A MnFe2O4/C nanocomposite as a novel theranostic agent in MRI, sonodynamic therapy and photothermal therapy of a melanoma cancer model. J Alloys Compd.

[120] Sheng Y, Beguin E, Nesbitt H, Kamila S, Owen J, Barnsley LC (2017). Magnetically responsive microbubbles as delivery vehicles for targeted sonodynamic and antimetabolite therapy of pancreatic cancer. J Control Release.

[121] Beguin E, Gray MD, Logan KA, Nesbitt H, Sheng Y, Kamila S (2020). Magnetic microbubble mediated chemo-sonodynamic therapy using a combined magnetic-acoustic device. J Control Release.

[122] Zhang Y, Xu Y, Sun D, Meng Z, Ying W, Gao W (2020). Hollow magnetic nanosystem-boosting synergistic effect between magnetic hyperthermia and sonodynamic therapy via modulating reactive oxygen species and heat shock proteins. Chem Eng J.

[123] Shen S, Wu L, Liu J, Xie M, Shen H, Qi X (2015). Core-shell structured Fe3O4@TiO2-doxorubicin nanoparticles for targeted chemo-sonodynamic therapy of cancer. Int J Pharm.

[124] Wang Z, Fu L, Zhu Y, Wang S, Shen G, Jin L (2021). Chemodynamic/photothermal synergistic therapy based on Ce-doped Cu-Al layered double hydroxides. J Mater Chem B.

[125] Gong Z, Dai Z (2021). Design and challenges of sonodynamic therapy system for cancer theranostics: from equipment to sensitizers. Adv Sci.

[126] Li W-P, Su C-H, Chang Y-C, Lin Y-J, Yeh C-S (2016). Ultrasound-induced reactive oxygen species mediated therapy and imaging using a Fenton reaction activable polymersome. ACS Nano.

[127] Hayes BT, Merrick MA, Sandrey MA, Cordova ML (2004). Three-MHz ultrasound heats deeper into the tissues than originally theorized. J Athl Train.

[128] Culjat MO, Goldenberg D, Tewari P, Singh RS (2010). A review of tissue substitutes for ultrasound imaging. Ultrasound Med Biol.

[129] Blomley MJK, Cooke JC, Unger EC, Monaghan MJ, Cosgrove DO (2001). Microbubble contrast agents: a new era in ultrasound. BMJ.

[130] Güvener N, Appold L, de Lorenzi F, Golombek SK, Rizzo LY, Lammers T (2017). Recent advances in ultrasound-based diagnosis and therapy with micro- and nanometer-sized formulations. Methods.

[131] Evertsson M, Kjellman P, Cinthio M, Andersson R, Tran TA, Zandt R (2017). Combined Magnetomotive ultrasound, PET/CT, and MR imaging of (68)Ga-labelled superparamagnetic iron oxide nanoparticles in rat sentinel lymph nodes *in vivo*. Sci Rep.

[132] O'Donnell M (2018). Magnetic nanoparticles as contrast agents for molecular imaging in medicine. Phys C Supercond Appl.

[133] Karwat P, Kujawska T, Lewin PA, Secomski W, Gambin B, Litniewski J (2016). Determining temperature distribution in tissue in the focal plane of the high (> 100 W/cm2) intensity focused ultrasound beam using phase shift of ultrasound echoes. Ultrasonics.

[134] Oh J, Feldman MD, Kim J, Condit C, Emelianov S, Milner TE (2006). Detection of magnetic nanoparticles in tissue using magneto-motive ultrasound. Nanotechnology.

[135] Evertsson M, Cinthio M, Kjellman P, Fredriksson S, Andersson R, Toftevall H (2015). *In vivo* magnetomotive ultrasound imaging of rat lymph nodes - a pilot study. IEEE Int Ultrason Symp..

[136] Bruno AC, Sampaio DR, Pavan TZ, Baffa O, Carneiro AA (2015). A hybrid transducer to evaluate stomach emptying by ultrasound and susceptometric measurements: an *in vivo* feasibility study. IEEE Trans Ultrason, Ferroelectr Freq Control.

[137] Qu M, Mehrmohammadi M, Truby R, Graf I, Homan K, Emelianov S (2014). Contrast-enhanced magneto-photo-acoustic imaging *in vivo* using dual-contrast nanoparticles. Photoacoustics.

[138] Mehrmohammadi M, Yoon KY, Qu M, Johnston KP, Emelianov SY (2010). Enhanced pulsed magneto-motive ultrasound imaging using superparamagnetic nanoclusters. Nanotechnology.

[139] Wang H, Huang C, Li M, editors (2019). Improved backward mode pulsed magnetomotive ultrasound via pre-magnetization of superparamagnetic iron oxide nanoparticles. IEEE Int Ultrason Symp.

[140] Fink M, Lyer S, Alexiou C, Rupitsch SJ, Ermert H (2019). Quantitative imaging of the iron-oxide nanoparticle concentration for magnetic drug targeting employing inverse magnetomotive ultrasound. Curr Dir Biomed Eng.

[141] Kim J, Oh J, Milner TE, Nelson JS (2007). Imaging nanoparticle flow using magneto-motive optical Doppler tomography. Nanotechnology.

[142] Mariappan L, Shao Q, Jiang C, Yu K, Ashkenazi S, Bischof JC (2016). Magneto acoustic tomography with short pulsed magnetic field for in-vivo imaging of magnetic iron oxide nanoparticles. Nanomed Nanotechnol Biol Med.

[143] Evertsson M, Cinthio M, Fredriksson S, Olsson F, Persson HW, Jansson T (2013). Frequency- and phase-sensitive magnetomotive ultrasound imaging of superparamagnetic iron oxide nanoparticles. IEEE Trans Ultrason, Ferroelectr Freq Control.

[144] Evertsson M, Kjellman P, Cinthio M, Fredriksson S, Zandt Rit, Persson HW Multimodal detection of iron oxide nanoparticles in rat lymph nodes using magnetomotive ultrasound imaging and magnetic resonance imaging IEEE Trans Ultrason, Ferroelectr Freq Control. 2014; 61(8): 1276-83.

[145] Sampaio DRT, Grillo FW, Bruno AC, Pavan TZ, Carneiro AAO (2016). A magneto-motive ultrasound platform designed for pre-clinical and clinical applications. Res Biomed Eng.

[146] Hadadian Y, Uliana JH, Carneiro AAO, Pavan TZ (2020). A Novel Theranostic Platform: Integration of Magnetomotive and Thermal Ultrasound Imaging with Magnetic Hyperthermia. IEEE Trans Biomed Eng.

[147] Rodrigues HF, Capistrano G, Bakuzis AF (2020). *In vivo* magnetic nanoparticle hyperthermia: a review on preclinical studies, low-field nano-heaters, noninvasive thermometry and computer simulations for treatment planning. Int J Hyperth.

[148] Liu S, Zhang R, Zheng Z, Zheng Y (2018). Electromagnetic-acoustic sensing for biomedical applications. Sensors.

[149] Sjöstrand S, Evertsson M, Thring C, Bacou M, Farrington S, Moug S (2019). Contrast-enhanced magnetomotive ultrasound imaging (CE-MMUS) for colorectal cancer staging: assessment of sensitivity and resolution to detect alterations in tissue stiffness. IEEE Int Ultrason Symp.

[150] Opieliński KJ, Pruchnicki P, Gudra T, Podgórski P, Kraśnicki T, Kurcz J (2013). Ultrasound transmission tomography imaging of structure of breast elastography phantom compared to US, CT and MRI. Arch Acoust.

[151] Opieliński KJ, Pruchnicki P, Gudra T, Podgórski P, Kurcz J, Kraśnicki T (2015). Imaging results of multi-modal ultrasound computerized tomography system designed for breast diagnosis. Comput Med Imaging Graph.

[152] Perlman O, Azhari H (2017). Ultrasonic computed tomography imaging of iron oxide nanoparticles. Phys Med Biol.

[153] Podaru GV, Chikan V, Prakash P (2016). Magnetic field induced ultrasound from colloidal superparamagnetic nanoparticles. J Phys Chem C.

[154] Carrey J, Connord V, Respaud M (2013). Ultrasound generation and high-frequency motion of magnetic nanoparticles in an alternating magnetic field: Toward intracellular ultrasound therapy?. Appl Phys Lett.

[155] Shoval A, Tepper M, Tikochkiy J, Gur LB, Markovich G, Keisari Y (2016). Magnetic nanoparticles-based acoustical detection and hyperthermic treatment of cancer, *in vitro* and *in vivo* studies. J Nanophotonics.

[156] Wei D, You-An S, Bi-Nan S, Ye-Wei G, Yan-Xia D, Guang-Ming X (2017). Reconstruction of internal temperature distributions in heat materials by ultrasonic measurements. Appl Therm Eng.

[157] Zhou C, Wang Y, Qiao C, Dai W (2016). Calibration method of an ultrasonic system for temperature measurement. PLOS ONE.

[158] Feng X, Gao F, Zheng Y (2015). A self-monitored theranostic platform based on nanoparticle hyperthermia therapy and alternating magnetic field induced thermoacoustic imaging. In: Oraevsky AA, Wang LV, Proc. SPIE 9323, Photons Plus Ultrasound: Imaging and Sensing.

[159] Piao D, Towner RA, Smith N, Chen WR (2013). Magnetothermoacoustics from magnetic nanoparticles by short bursting or frequency chirped alternating magnetic field: A theoretical feasibility analysis. Med Phys.

[160] Kłosowski G, Rymarczyk T, Kania K, Świć A, Cieplak T (2020). Maintenance of industrial reactors supported by deep learning driven ultrasound tomography. Eksploat Niezawodn.

[161] Bush N, Healey A, Shah A, Box G, Kirkin V, Eccles S (2020). Theranostic attributes of acoustic cluster therapy and its use for enhancing the effectiveness of liposomal doxorubicin treatment of human triple negative breast cancer in mice. Front Pharmacol.

[162] Kuhn J, Papanastasiou G, Tai C-W, Moran CM, Jansen MA, Tavares AAS (2020). Tri-modal imaging of gold-dotted magnetic nanoparticles for magnetic resonance imaging, computed tomography and intravascular ultrasound: an *in vitro* study. Nanomedicine.

[163] Zhang K, Li P, Chen H, Bo X, Li X, Xu H (2016). Continuous cavitation designed for enhancing radiofrequency ablation via a special radiofrequency solidoid vaporization process. ACS Nano.

[164] Allen SP, Steeves T, Fergusson A, Moore D, Davis RM, Vlaisialjevich E (2019). Novel acoustic coupling bath using magnetite nanoparticles for MR-guided transcranial focused ultrasound surgery. Med Phys.

[165] Huang K-W, Chieh J-J, Yeh C-K, Liao S-H, Lee Y-Y, Hsiao P-Y (2017). Ultrasound-induced Magnetic imaging of tumors targeted by biofunctional magnetic nanoparticles. ACS Nano.

[166] Hingot V, Errico C, Heiles B, Rahal L, Tanter M, Couture O (2019). Microvascular flow dictates the compromise between spatial resolution and acquisition time in Ultrasound Localization Microscopy. Sci Rep.

[167] Malhotra N, Lee J-S, Liman RAD, Ruallo JMS, Villaflores OB, Ger T-R (2020). Potential toxicity of iron oxide magnetic nanoparticles: a review. Molecules.

[168] Karlsson HL, Cronholm P, Gustafsson J, Möller L (2008). Copper oxide nanoparticles are highly toxic: a comparison between metal oxide nanoparticles and carbon nanotubes. Chem Res Toxicol.

[169] Molcan M, Kaczmarek K, Kubovcikova M, Gojzewski H, Kovac J, Timko M (2020). Magnetic hyperthermia study of magnetosome chain systems in tissue-mimicking phantom. J Mol Liq.

[170] Nima ZA, Watanabe F, Jamshidi-Parsian A, Sarimollaoglu M, Nedosekin DA, Han M (2019). Bioinspired magnetic nanoparticles as multimodal photoacoustic, photothermal and photomechanical contrast agents. Sci Rep.

